# Self-organization of vascularized muscle from bovine embryonic stem cells

**DOI:** 10.1038/s41467-026-76569-2

**Published:** 2026-09-02

**Authors:** Marina Sanaki-Matsumiya, Yuya Sanaki, Casandra Villava, Luca Rappez, Nicola Gritti, Fumio Nakaki, James Sharpe, Kristina Haase, Jun Wu, Miki Ebisuya

**Affiliations:** 1 European Molecular Biology Laboratory, EMBL Barcelona, C/ Dr. Aiguader, Barcelona, Spain; 2 Life Science Center for Survival Dynamics, Tsukuba Advanced Research Alliance (TARA), University of Tsukuba, Ibaraki, Japan; 3 Department of Molecular Biology, University of Texas Southwestern Medical Center, Dallas, TX USA; 4 Hamon Center for Regenerative Science and Medicine, University of Texas Southwestern Medical Center, Dallas, TX USA; 5 Cecil H. and Ida Green Center for Reproductive Biology Sciences, University of Texas Southwestern Medical Center, Dallas, TX USA; 6 Cluster of Excellence Physics of Life, TU Dresden, Dresden, Germany

**Keywords:** Stem-cell differentiation, Tissue engineering, Musculoskeletal development

## Abstract

Cultured beef offers a promising alternative to traditional meat. While adult stem cells are commonly used as the cell source for cultured beef, their proliferation and differentiation capacities are limited. Current manufacturing processes for cultured beef steaks often require separate preparation of multiple cell types and their intricate assembly. In this study, we propose and report the co-induction of muscle, neural, and endothelial cells from bovine embryonic stem cells (ESCs), accompanied by the formation of tissue-like structures in two- and three-dimensional cultures. Bovine myocytes are induced in a stepwise manner through the induction of presomitic mesoderm (PSM) from bovine ESCs. Muscle fibers with sarcomeres appear within 15 days, displaying calcium oscillations responsive to inputs from co-induced bovine neurons. Bovine endothelial cells are also co-induced via PSM, forming uniform vessel networks inside tissues. Our serum-free, rapid co-induction protocols represent a milestone toward self-organizing beef steaks with integrated vasculature and innervation.

## Abstract (web summary)

Here they developed self-organizing bovine tissues containing skeletal muscle, neural, and endothelial cells derived from bovine embryonic stem cells, with potential applications in cultured beef production.

## Introduction

Cultured meat, also referred to as cultivated meat, cell-based meat, lab-grown meat, or clean meat, is produced through culturing animal skeletal muscle cells in vitro^1–4^. Cultured meat has the potential to address ethical, environmental, and health concerns associated with conventional meat production from livestock.

Current practices in cultured beef production predominantly rely on myosatellite cells and mesenchymal stem cells (MSCs) isolated from adult bovine tissue biopsy and bone marrow samples^4–6^. The proliferation capacity of these adult stem cells diminishes over long-term culture. Although they can be immortalized^7^, the genetic modifications required for immortalization may present a challenge in obtaining food authorization. The differentiation capacity of adult stem cells is also limited. For instance, myosatellite cells are committed to muscle fate; although MSCs can differentiate into osteoblasts, adipocytes, and chondrocytes in addition to myocytes, the differentiation trajectory of MSCs is still restricted and dependent on the tissue source from which they are isolated. By contrast, pluripotent stem cells, namely embryonic stem cells (ESCs) and induced pluripotent stem cells (iPSCs) proliferate indefinitely, offering a more stable cell source for cultured meat. Pluripotent stem cells can also differentiate into cells of all three germ layers: mesoderm, ectoderm, and endoderm. Unmodified ESCs are more favorable for food production than iPSCs that need genetic reprogramming. The derivation of stable bovine ESCs was historically challenging but recently achieved^8^, and a large-scale culture of bovine ESCs in stirred bioreactors has been reported^9^.

Another challenge in the cultured meat field is how to produce structured steaks instead of ground meat. The incorporation of diverse cell types within the muscle tissue holds the key to more accurate recreation of steaks^10,11^. For in vitro tissues to keep growing, oxygen and nutrient supply through blood vessels or alternative porous structures is also crucial^12^. The current manufacturing strategy for cultured beef steaks involves the separate preparation of distinct cell types followed by their assembly. This assembly process necessitates advanced engineering techniques, such as bioprinting purified cells with a bioink, co-culturing cells on edible porous scaffolds, and stacking myocyte-laden hydrogel modules^13–16^. Additionally, electrical or mechanical stimuli are often applied to facilitate muscle maturation and improve the texture^16–21^. While these engineering approaches are promising and evolving rapidly, they add extra manufacturing steps to cultured steak production. A potential alternative approach is utilizing pluripotent stem cells and leveraging their differentiation and self-organization abilities that have been demonstrated by the development of a variety of complex organoids in recent years^22–28^.

In this study, we utilized bovine ESCs to co-induce skeletal muscle, neural, and endothelial cells, fostering the formation of muscle tissues with innervation and vascularization.

## Results

### Induction of PSM and muscle fibers from bovine ESCs

**Fig. 1:**
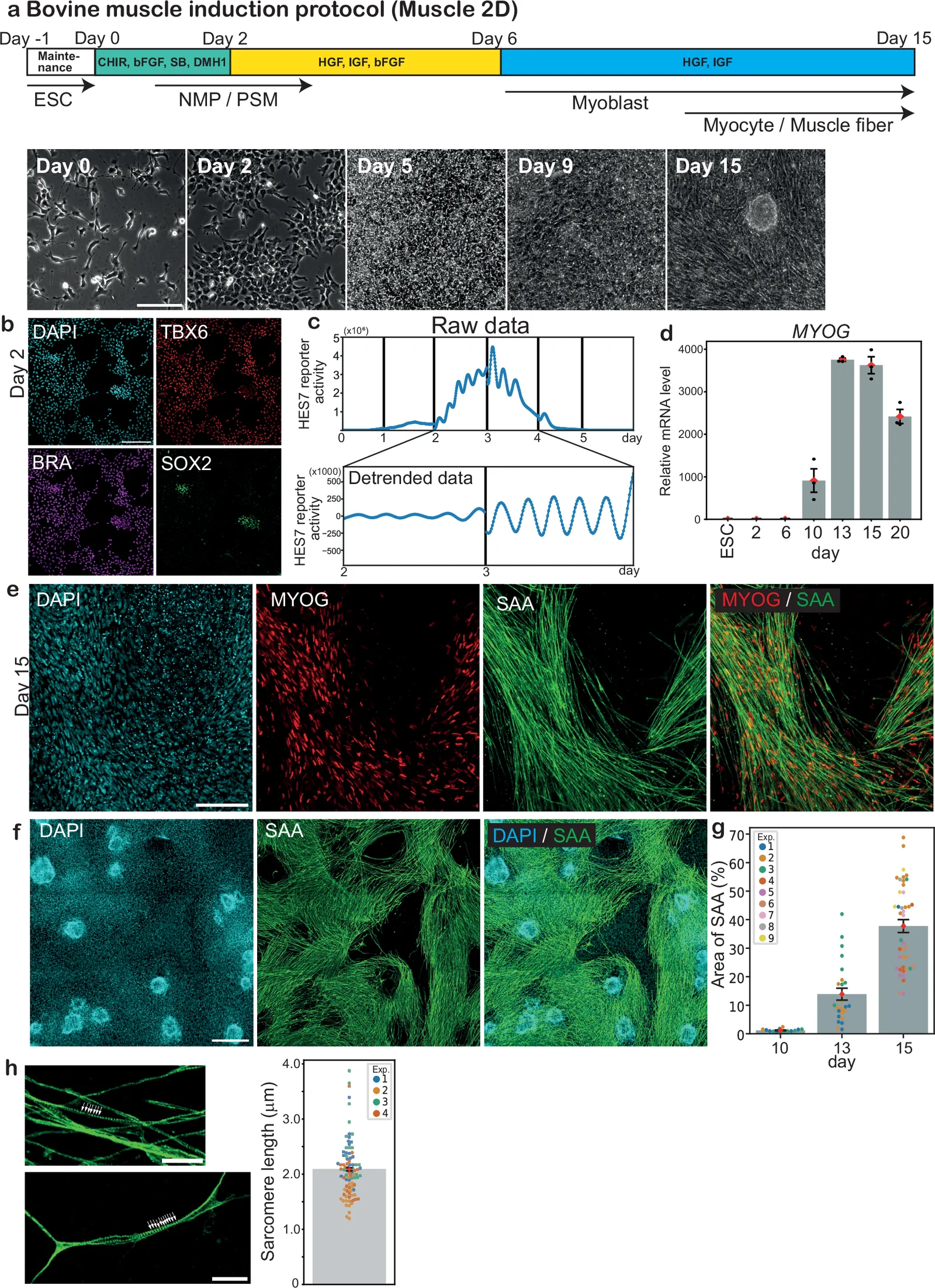
Induction of PSM and muscle fibers from bovine ESCs. **a** Induction protocol for bovine myocytes and muscle fibers from bovine ESCs. PSM cells were initially induced from ESCs using CHIR, bFGF, SB, and DMH1. Then myocytes were induced from PSM using HGF, IGF, and bFGF. Maintenance: Maintenance medium, ESC: Embryonic stem cell, NMP: Neuromesodermal progenitor, PSM: Presomitic mesoderm. Typical bright field images are also shown (bottom). **b** IHC images of the day-2 culture. TBX6 is a PSM marker. SOX2- and BRA-double positive cells are NMPs. 3 independent experiments showed similar staining patterns. **c** The oscillatory activity of the HES7 promoter-luciferase reporter in PSM. Raw (top) and detrended (bottom) signals. The vertical lines indicate the timings of medium change, which resets the oscillation phase. 3 independent experiments showed similar oscillation patterns. **d** qRT-PCR quantification of *MYOG* expression. The average value of ESC sample was set to 1. Mean ± SEM. *N* = 3 dishes from 3 independent experiments. **e**,**f** IHC images of the day-15 cultures. MYOG and SAA are myocyte and muscle fiber markers, respectively. 7 independent experiments showed similar staining patterns. **g** Percentage of the SAA-positive area in the field of view. Mean ± SEM. *N* = 14 images from 3 independent experiments (day 10), 25 images from 3 independent experiments (day 13), and 42 images from 9 independent experiments (day 15). **h** Sarcomeric structures on SAA-positive muscle fibers (arrowheads in the left pictures) on day 15. The sarcomere length was defined as the distance between intensity peaks (right). Mean ± SEM. *N* = 88 sarcomere fibers from 4 independent experiments. **g**,**h** Dot colors indicate individual experiments. Scale bars: 150 µm (**a**,**b**,**e**), 300 µm (**f**), and 30 µm (**h**). Microscopes: IX73 (**a**) and FV3000 confocal (**b**,**e**,**f**,**h**). Source data are provided as a Source Data file.

We first established a protocol to induce muscle fibers from bovine ESCs (Fig. 1a and Supplementary Fig. 1). As skeletal muscle is generated from presomitic mesoderm (PSM) through transient structures of somites and dermomyotomes in embryonic development^29^, our stepwise protocol comprised the induction of PSM followed by the induction of myoblasts and myocytes. Based on the PSM induction protocol we previously reported^30–33^, bovine ESCs were treated with CHIR (WNT signaling activator), bFGF, SB431542 (TGFb signaling inhibitor), and DMH1 (BMP signaling inhibitor) for 2 days. The expression levels of pluripotency markers, *NANOG*, *OCT4*, and *SOX2*, decreased before day 2 (Supplementary Fig. 2a) while those of the mesodermal marker *BRACHYURY* (*BRA*, also known as *T*) and the PSM marker *TBX6* increased (Supplementary Fig. 2b). Most cells were TBX6-positive on day 2, suggesting efficient PSM induction (Fig. 1b). Since the remarkable characteristic of PSM cells is the segmentation clock, the oscillatory gene expressions that regulate the timing of somite formation, we monitored the expression pattern of *HES7*, a core gene of the molecular oscillator^34,35^. A HES7 promoter-luciferase reporter^30–33^ showed an elevated expression around day 2 (Fig. 1c, top), simultaneously displaying clear oscillations of the segmentation clock (Fig. 1c, bottom). Consistent with the previous report^30^, the oscillation period of the bovine segmentation clock was ~4 h. These results confirmed the efficient induction of bovine PSM from bovine ESCs.

To further induce skeletal muscle from bovine PSM, we adapted the muscle induction protocol previously reported for mouse and human cells^36–38^. Bovine PSM cells were treated with HGF, IGF, and bFGF for 4 days before further treatment with HGF and IGF for 9 days (Fig. 1a). The expression levels of myoblast markers, *MYF5* and *MYOD1*, increased around day 6 (Supplementary Fig. 2c). The expressions of myocyte markers, *MYOG, MYF6*, *MYH7*, and *MYH4*, were induced around day 10 (Fig. 1d; Supplementary Fig. 2d). The MYOG- and sarcomeric α-actinin (SAA)-positive muscle fibers first appeared around day 10 and peaked around day 14 (Fig. 1e–g). These bovine muscle fibers displayed sarcomeric structures, and their average sarcomere length was 2.1 µm (Fig. 1h), similar to previously reported values for in vivo bovine skeletal muscles^39,40^. The ~15-day duration of bovine myocytes and muscle fiber induction was slightly shorter than the one described in the human skeletal muscle induction protocol^37^, potentially reflecting the faster pace of bovine embryonic development compared with human^30^. These results collectively established the induction protocol for bovine myocytes and muscle fibers from bovine PSM. Note that the protocol does not require serum.

### Co-induction of bovine muscle and neurons

**Fig. 2:**
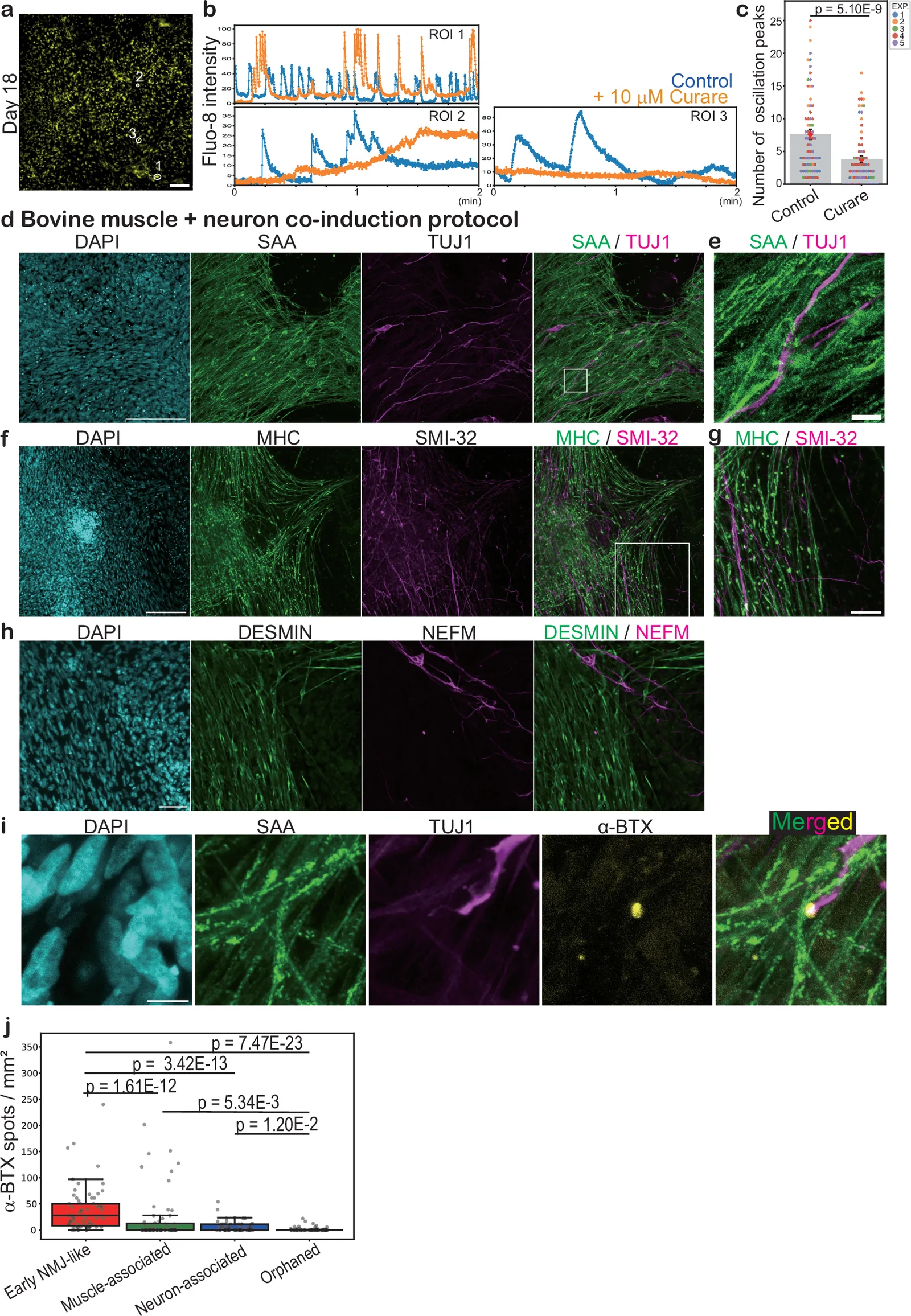
Co-induction of bovine muscle and neurons. **a** Fluo-8 calcium signal of the day-18 culture of the muscle 2D protocol. **b** Calcium level fluctuations in the absence (blue) and presence (orange) of 10 µM curare, an acetylcholine receptor blocker. Regions of interest (ROIs) are marked in (**a**). **c** Quantification of the total number of calcium oscillation peaks within 2 min in the absence and presence of curare. Mean ± SEM. *N* = 71 ROIs (the same ROIs between Control and Curare) from 5 independent experiments. *P*-value is from two-sided paired *t*test. Dot colors indicate individual experiments. **d–i** IHC images of the day-15 cultures. TUJ1, SMI-32, and NEFM are neuronal markers. SAA, MHC, and DESMIN are skeletal muscle markers. α-BTX is a neuromuscular junction (NMJ) marker. Four to twenty four independent experiments showed similar staining patterns. **e**,**g** Enlarged images of (**d**) and (**f**), respectively. **j** Classification of α-BTX-reactive spots based on their proximity to muscle fibers and neurons. Spot identification and classification were performed as shown in Supplementary Fig. 3c, d, and the number of spots in each category was normalized to the corresponding image area. Boxplots indicate the median, 25th percentile, and 75th percentile. Whiskers extend to the minimum and maximum non-outlier values. *N* = 67 images from 20 independent experiments were analyzed. Following a significant Friedman test, two-sided pairwise comparisons were performed using Conover’s post hoc test with Bonferroni-adjusted *p*-values. Adjusted *p*-values < 0.05 are shown in the graph. Scale bars: 150 µm (**a**,**d**,**f**), 50 µm (**g**,**h**), and 10 µm (**e**,**i**). Microscopes: Thunder (**a**), FV3000 confocal (**f**,**g**), and LSM980 (**b**,**e**,**h**,**i**). Source data are provided as a Source Data file.

The induced bovine myocytes and muscle fibers displayed spontaneous fluctuations of intracellular calcium levels (Fig. 2a, b; Supplementary Movie 1). The frequency and amplitude of these calcium transients varied among cells, potentially reflecting differences in cell density and maturation state. The calcium pulses were effectively suppressed by the treatment with curare (Fig. 2b, c; Supplementary Fig. 3a; Supplementary Movie 1), an acetylcholine receptor blocker that is used to inhibit neuromuscular junctions (NMJs)^28,41,42^. This suggested the presence of early NMJ-like structures connecting skeletal muscles and neurons. Indeed, we detected TUJ1-positive neurons intermingling with SAA-positive muscle fibers in the day-15 muscle culture (Fig. 2d, e). The expression of the neuronal marker *SOX2* also increased around day 10–13 (Supplementary Fig. 2a). Moreover, staining for more mature neurofilament markers (SMI-32 and NEFM), together with additional muscle markers (MHC and DESMIN), revealed similar patterns of aligned neurons and muscle fibers (Fig. 2f–h). These neurons may be, at least in part, spinal neurons derived from neuromesodermal progenitors (NMPs), as the day-2 PSM culture contained a fraction of SOX2- and BRA-double-positive NMPs (Fig. 1b; Supplementary Fig. 1b). We further stained with the NMJ marker α-bungarotoxin (α-BTX), which revealed punctate signals (Fig. 2i; Supplementary Fig. 3b). Although these puncta exhibited a rounded, immature morphology, they were frequently located at sites where muscle fibers and neurons were in close proximity, suggesting that they are early NMJ-like structures (Fig. 2j and Supplementary Fig. 3c, d). Together, these results suggest that neurons co-induced from bovine ESCs may transmit signals to skeletal muscle through early NMJ-like structures. The stimulation from neurons may be beneficial for muscle maturation, potentially offering an alternative to the external electrical and mechanical stimuli currently employed in the muscle bioengineering field^16–21^.

### Co-induction of bovine muscle and endothelial cells

**Fig. 3:**
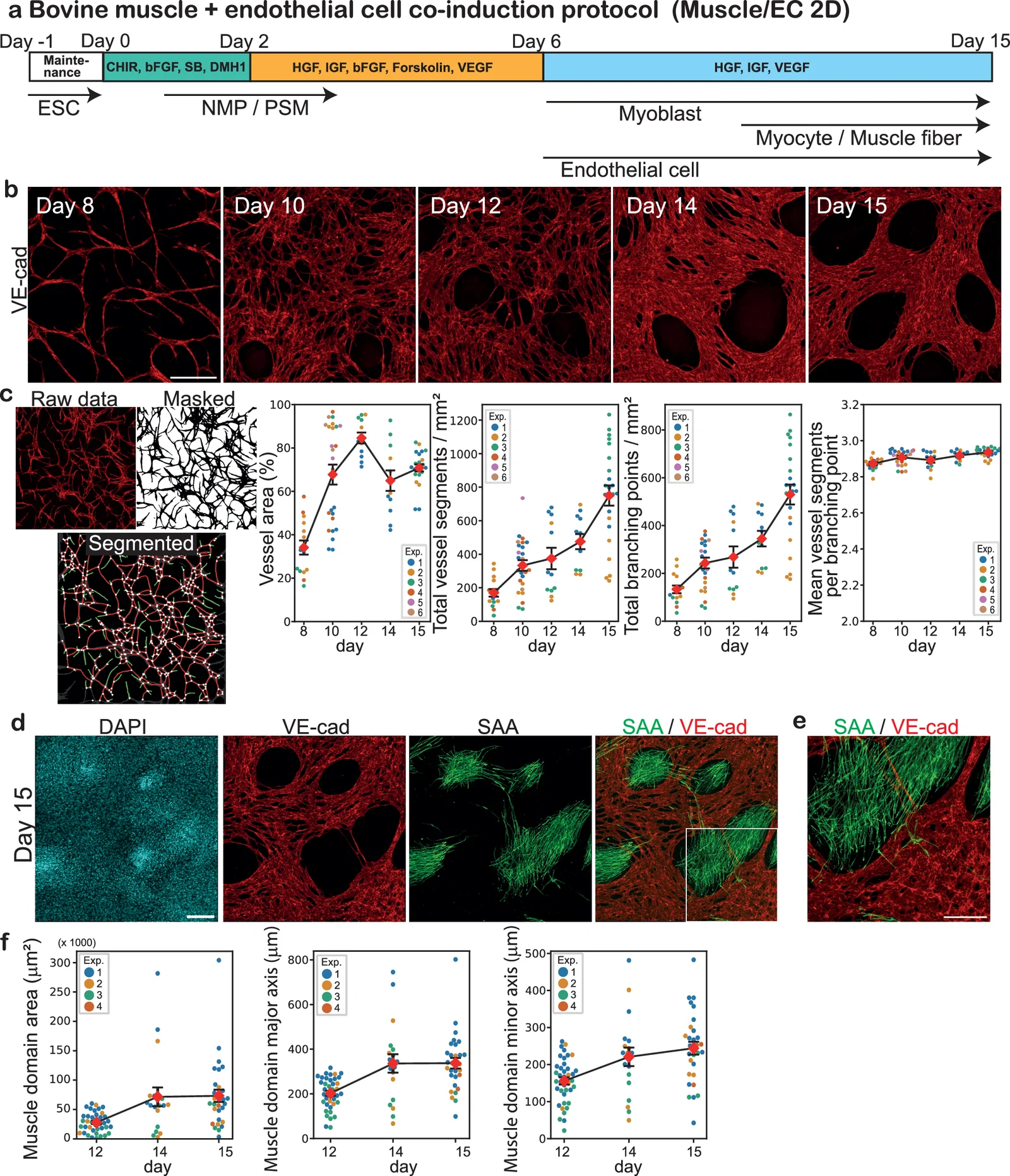
Co-induction of bovine muscle and endothelial cells. **a** Co-induction protocol for bovine muscle fibers and endothelial cells. Forskolin and VEGF were added to induce endothelial cells from bovine PSM cells. **b** IHC images over the time course. VE-cad is an endothelial cell marker. 3-6 independent experiments showed similar staining patterns. **c** Characterization of the endothelial cell networks using VascuMap. After the IHC images were masked and segmented, the vessel segments (red edges) and branching points (white nodes) were identified and quantified using the connectivity analysis pipeline. The vessel segments that do not connect two points (green edges) were not counted. The vessel area, the total number of vessel segments, the total number of branching points, and the mean number of vessel segments per branching point were quantified. Mean ± SEM. *N* = 15 images from 4 independent experiments (day 8), 25 images from 6 independent experiments (day 10), 12 images from 3 independent experiments (day 12), 12 images from 3 independent experiments (day 14), and 23 images from 3 independent experiments (day 15). **d** IHC images of the day-15 culture showing endothelial and skeletal muscle domains. 15 independent experiments showed similar staining patterns. **e** Enlarged image of (**d**). **f** Size of skeletal muscle domains. The area, major axis length, and minor axis length of VE-cad-negative domains were quantified. Mean ± SEM. *N* = 39 images from 3 independent experiments (day 12), 19 images from 3 independent experiments (day 14), and 30 images from 4 independent experiments (day 15). **c**,**f** Dot colors indicate individual experiments. Scale bars: 300 µm (**b**,**d**) and 150 µm (**e**). Microscope: FV3000 confocal. Source data are provided as a Source Data file.

The lack of blood vessels is a major challenge that currently hampers the development of large, mature organoids in the stem cell biology field. We therefore sought to develop a protocol to co-induce skeletal muscle and endothelial cells from bovine ESCs (Fig. 3a), which in the future may allow perfusion. As blood vessels are first formed by endothelial cells, we prioritized the induction of bovine endothelial cells. Note that while endothelial cells arise primarily from lateral plate mesoderm in embryonic development^43,44^, a contingent of endothelial cells are derived from PSM and somites^44–49^. We thus hypothesized that endothelial cells and myocytes could be co-induced from the same PSM cell population. To test this, we integrated our bovine muscle induction protocol with established protocols for human endothelial cells^50–52^, by treating PSM cells with the angiogenic factors VEGF and forskolin (cAMP signaling activator) together with the skeletal muscle induction factors HGF, IGF, and bFGF (Fig. 3a). The BMP inhibitor DMH1 that prevents lateral plate mesoderm differentiation was kept in the protocol. VEGF and forskolin were added to the bovine PSM culture on day 2. The elongated cells, positive for the endothelial marker VE-cadherin (VE-cad, also known as CDH5), appeared around day 8 and peaked around day 14 (Fig. 3b). The induced bovine endothelial cells formed interconnected vessel-like networks: the numbers of vessel segments and branching points increased during days 8–15 (Fig. 3c). The endothelial networks and muscle fibers tended to form small individual domains (Fig. 3d, e). The skeletal muscle domains encased by endothelial cells measured 200–300 µm in diameter on day 15 (Fig. 3f), which surpassed the intercapillary distance of approximately 100 µm in tissues^53^, yet remained within a comparable range. TUJ1-positive neurons were also co-induced under this protocol (Supplementary Fig. 4). These results demonstrated the co-induction of skeletal muscle, neurons, and endothelial cells from bovine PSM and the self-organization of vascular networks in two-dimensional (2D) cultures.

### Induction of bovine muscle fibers in 3D cultures

**Fig. 4:**
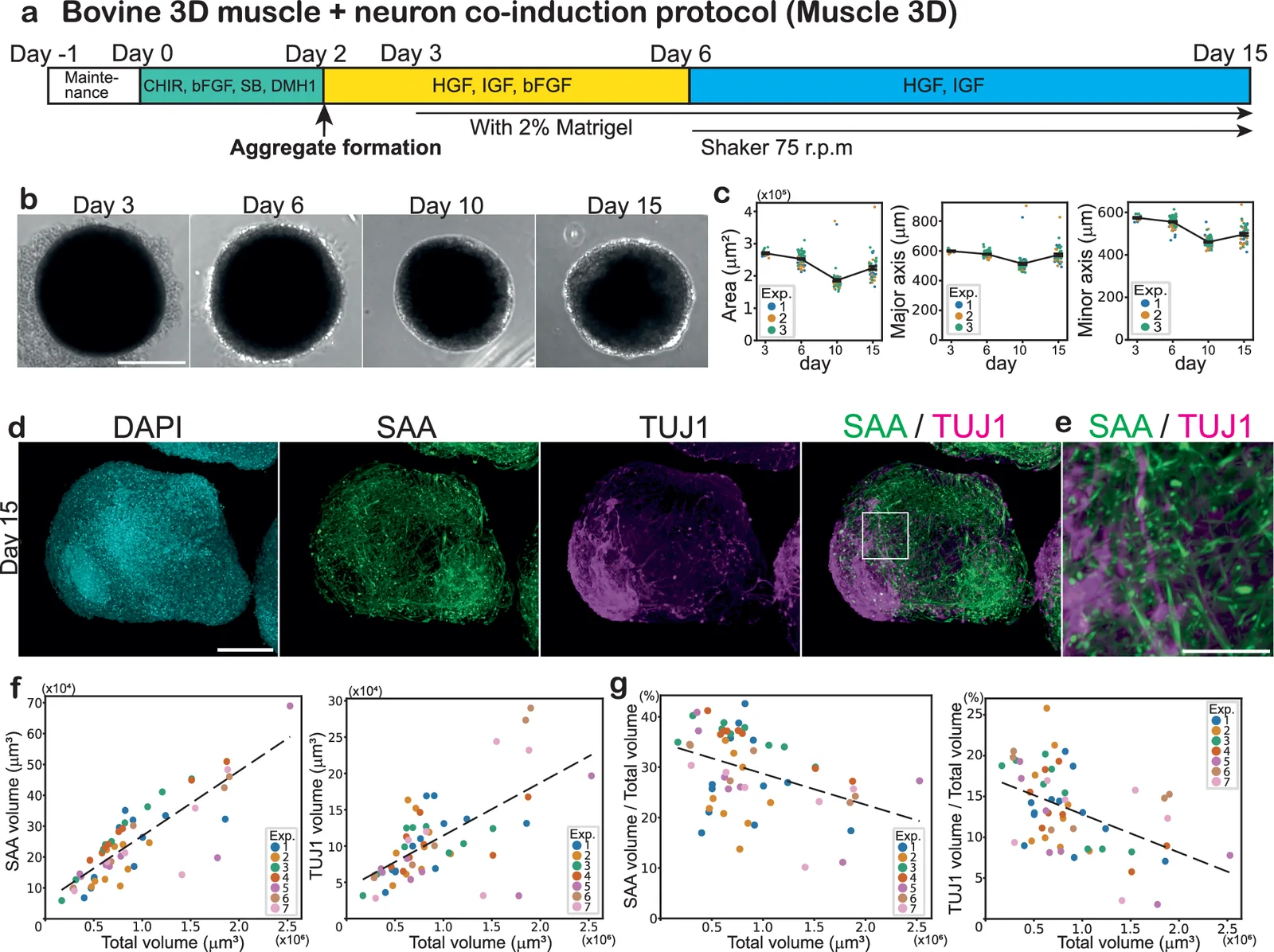
Induction of bovine muscle fibers in 3D cultures. **a** 3D induction protocol for bovine muscle fibers and neurons. The aggregate of 20,000 bovine PSM cells was made on day 2. Matrigel was added to the medium on day 3. Aggregates were cultured on a rotary shaker after day 6. **b**,**c** Characterization of aggregates over the time course. The area, major axis length, and minor axis length of the aggregates were quantified using 2D bright field images. Mean ± SEM. *N* = 15 (day 3), 93 (day 6), 90 (day 10), and 48 (day 15) aggregates from 3 independent experiments. **d** IHC images of the day-15 aggregate. SAA and TUJ1 are muscle fiber and neuronal markers, respectively. 16 independent experiments showed similar staining patterns. **e** Enlarged image of (**d**). **f**,**g** Effects of aggregate size on cell fates. The aggregates were made from 10,000 to 30,000 PSM cells on day 2, and the percentages of the SAA- and TUJ1-positive volumes were quantified in the day-15 aggregates. *N* = 57 aggregates from 7 independent experiments. **c**,**f**,**g** Dot colors indicate individual experiments. Scale bars: 300 µm (b), 150 µm (**d**) and 50 µm (**e**). Microscopes: IX73 (**b**) and Light-sheet (**d**,**e**). Source data are provided as a Source Data file.

With the ultimate goal of self-organizing beef steaks in mind, we applied our bovine ESC differentiation protocols to three-dimensional (3D) cultures. We developed a protocol to induce bovine muscle fibers in 3D cell aggregates (Fig. 4a). Bovine PSM cells were induced according to our 2D protocol, and on day 2, a 3D aggregate was created out of 20,000 PSM cells (Fig. 4b), which contained a small fraction of NMPs. The 2% Matrigel was added to the medium to bolster the structural integrity of aggregates: without Matrigel, the aggregates gradually fell apart, and a higher concentration of Matrigel triggered the separation of two aggregate domains (Supplementary Fig. 5). The PSM cell aggregates were treated with the skeletal muscle-inducing cocktail, and after day 6, they were cultured on a rotary shaker that facilitated the diffusion of oxygen and nutrients (Fig. 4a). The aggregates were spherical and ~600 µm in diameter (Fig. 4c), and the size did not dramatically change over the time course, probably reflecting a declined cell proliferation during the muscle maturation phase. SAA-positive muscle fibers were induced around day 10 (Fig. 4d and Supplementary Movie 2). Consistent with the results from our 2D cultures, TUJ1-positive neurons were co-induced in the aggregates, forming neural domains next to muscle domains (Fig. 4d, e and Supplementary Movie 2). To assess the effects of aggregate size on cell fates, we prepared diverse sizes of aggregates by changing the initial PSM cell number. The proportions of the skeletal muscle and neural domains gradually decreased as the aggregate size increased (Fig. 4f, g). All samples (*N* = 57) formed aggregates and exhibited an SAA/DAPI staining ratio of at least 10% (Fig. 4g), supporting the reproducibility of the protocol. Together, these results established the induction protocol for a bovine 3D tissue comprising skeletal muscle and neurons from bovine ESCs.

### Co-induction of bovine muscle and endothelial networks in 3D

**Fig. 5:**
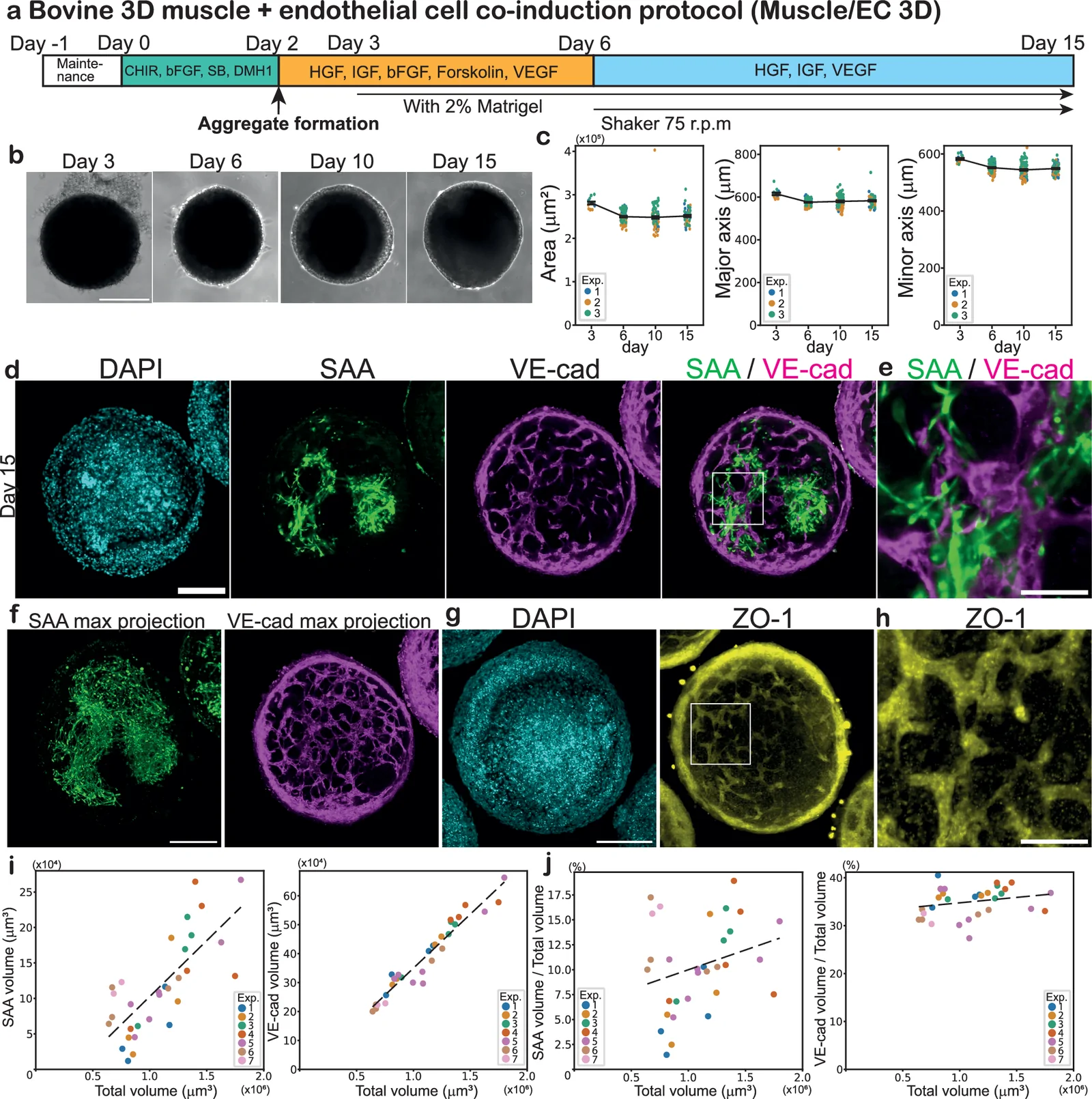
Co-induction of bovine muscle and endothelial networks in 3D cultures. **a** 3D co-induction protocol for bovine muscle fibers and endothelial cell networks. The aggregate of 20,000 bovine PSM cells was made on day 2. Forskolin and VEGF were added to induce endothelial cells from PSM cells. **b**,**c** Characterization of aggregates over the time course. The area, major axis length, and minor axis length of the aggregates were quantified using 2D bright field images. Mean ± SEM. *N* = 15 (day 3), 94 (day 6), 93 (day 10), and 65 (day 15) aggregates from 3 independent experiments. **d–h** IHC images of the day-15 aggregates. **d** SAA and VE-cad are muscle fiber and endothelial cell markers, respectively. 15 independent experiments showed similar staining patterns. See also Supplementary Movie 3. **e** Enlarged image of (**d**). **f** Max projection images of d. The max projection of SAA used all Z-stacks. The max projection of VE-cad used the Z-stacks within the 40 μm range around the central slice. **g** ZO-1 is a tight junction marker. Max projection used the Z-stacks within the 40 μm range around the central slice. 3 independent experiments showed similar staining patterns. See also Supplementary Movie 4. **h** Enlarged image of g. **i**,**j** Effects of aggregate size on cell fates. The aggregates were made from 10,000-30,000 PSM cells on day 2, and the percentages of the VE-cad- and SAA-positive volumes were quantified in the day-15 aggregates. Mean ± SEM. *N* = 31 aggregates from 7 independent experiments. **c**,**i**,**j** Dot colors indicate individual experiments. Scale bars: 300 µm (**b**), 150 µm (**d**,**f**,**g**), and 50 µm (**e**,**h**). Microscopes: IX73 (**b**) and Light-sheet (**d–h**). Source data are provided as a Source Data file.

Finally, we developed a protocol to co-induce muscle fibers, neurons, and endothelial cell networks from bovine ESCs in 3D cultures (Fig. 5a). The aggregates of bovine PSM cells were created on day 2, and then treated with the induction cocktail for skeletal muscle and endothelial cells. The aggregate size was again ~600 µm in diameter over the entire time course (Fig. 5b, c). VE-cad-positive endothelial cells emerged around day 10, forming interconnected vessel-like networks adjacent to the domains of muscle fibers inside the aggregates (Fig. 5d–f and Supplementary Movie 3). ZO-1 staining unveiled similarly connected networks, suggesting the presence of tight junctions in the endothelial networks (Fig. 5g, h and Supplementary Movie 4). Note that the endothelial networks displayed a regular pattern throughout an aggregate, penetrating deeply into the tissue (Supplementary Movie 3, 4). The abundant and relatively uniform distribution of vasculature will be advantageous for facilitating oxygen and nutrient delivery throughout the muscle aggregates, although using this network for intraluminal perfusion remains to be seen. TUJ1-positive neurons were also co-induced in the aggregates, forming neural domains (Supplementary Fig. 6a). The endothelial network formation depended on the induction protocols: when PSM cells were induced without the BMP inhibitor DMH1, the subsequent endothelial cells did not form uniform networks, and the shape of the aggregates was no longer spherical (Supplementary Fig. 6b and Supplementary Movie 5). Unlike the aggregates that lack endothelial cells (Fig. 4g), the proportions of the muscle domain and the endothelial networks slightly increased or remained constant as the aggregate size increased (Fig. 5i, j). These results underscored the feasibility of generating vascularized skeletal muscle tissues from bovine ESCs.

### Cell types induced from bovine ESCs

**Fig. 6:**
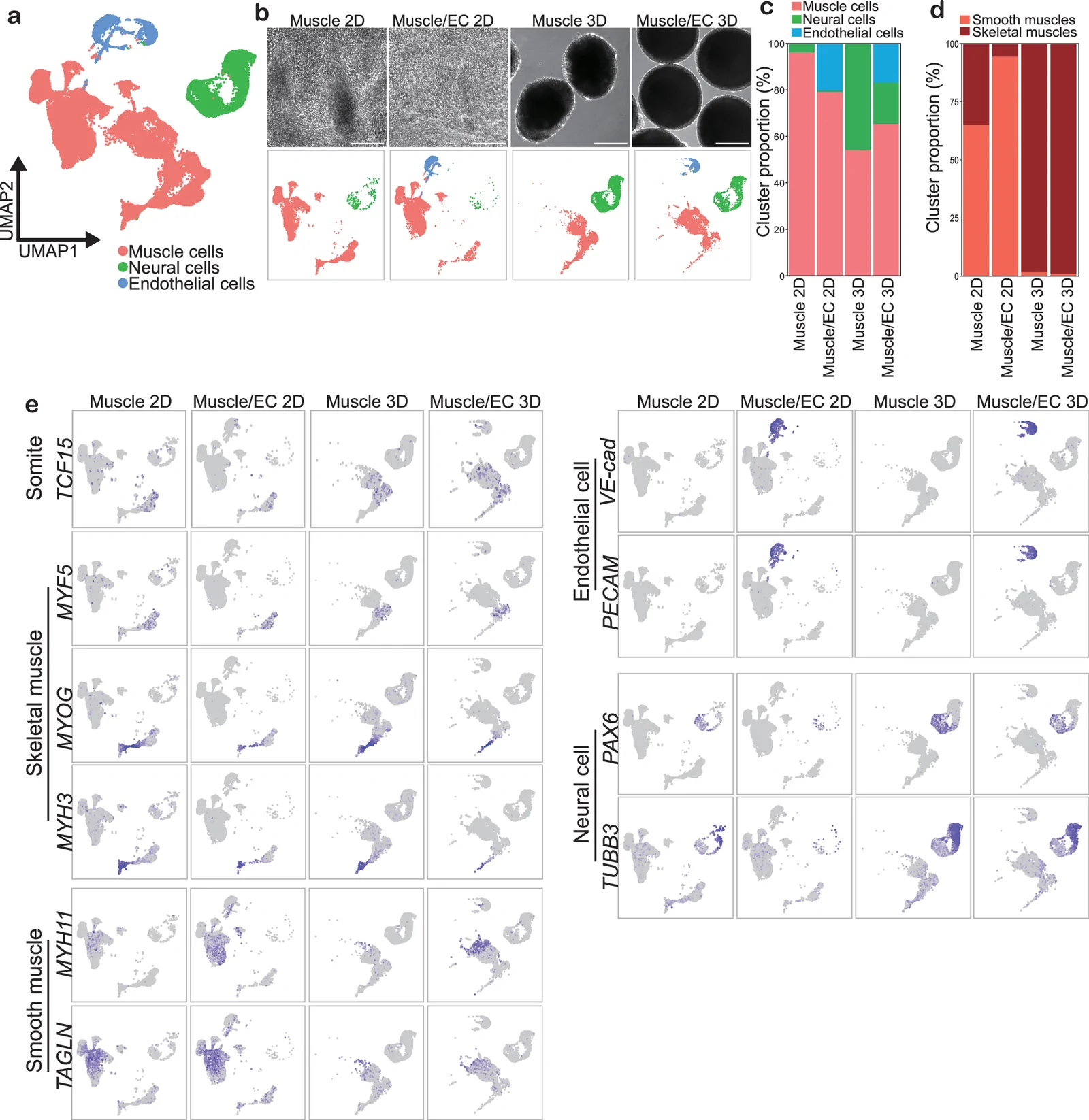
Single-cell RNA sequencing analyses of individual induction protocols. **a** Uniform manifold approximation and projection (UMAP) plot of the integrated data from 4 samples shown in (**b**) colored by 3 clusters. **b** Example bright field images of the 4 samples corresponding to individual induction protocols on day 15 (top). Scale bars: 300 µm. Split UMAP plots of individual samples (bottom). *N* = 8562 (Muscle 2D protocol), 10294 (Muscle/EC 2D protocol), 8247 (Muscle 3D protocol; 30 aggregates), and 7676 (Muscle/EC 3D protocol; 30 aggregates) cells from a single experiment. EC: Endothelial cell. Statistic information for the scRNA-seq samples is in Supplementary Data 3. **c** Proportions of the muscle, neural, and endothelial cell clusters in individual samples. **d** Proportions of the 2 muscle subclusters in individual samples. **e** UMAP plots colored by the expression of selected marker genes for somite, skeletal muscle, smooth muscle, endothelial, and neural cells. Source data are provided as a Source Data file.

Our single-cell RNA sequencing (scRNA-seq) analyses validated the presence of muscle and neural cell clusters (Fig. 6a and Supplementary Figs. 7, 8). The endothelial cell (EC) cluster was exclusively detected when using the EC co-induction protocols (Fig. 6b, c). Muscle cells were further classified into skeletal and smooth muscle subclusters (Fig. 6d and Supplementary Fig. 8a–e), with the 2D induction protocols yielding higher proportions of smooth muscle compared to the 3D protocols. Consistently, staining for the smooth muscle marker α-smooth muscle actin (α-SMA) revealed abundant smooth muscle domains in 2D cultures that were spatially distinct from MYOG- or SAA-positive skeletal muscle domains (Supplementary Fig. 9), suggesting that the skeletal muscle-rich 3D cultures are more suitable for cultured beef applications than the 2D cultures. Both skeletal and smooth muscle subclusters contained fibroblast-like cells (Supplementary Fig. 8f). While mature fat cells were not explicitly identified, several adipocyte progenitor markers were detected, particularly under the EC co-induction protocols (Supplementary Fig. 8g). Similarly, the expression of pericyte and osteoblast markers was more effectively induced under the EC co-induction protocols (Supplementary Fig. 8h, i). The pericyte marker NG2 also exhibited punctate staining signals in contact with VE-cad-positive endothelial cells (Supplementary Fig. 9c).

The neural cell cluster was more efficiently induced under the 3D protocols compared to the 2D protocols (Fig. 6b, c and Supplementary Fig. 10a–c). Neural cells were further classified into neural progenitors and mature neural cell subclusters, with both subclusters also expressing glial cell markers (Supplementary Fig. 10d–h), suggesting the heterogeneous nature of neural subtypes. The detection of spinal neuron markers supported the NMP origin of the induced neurons (Supplementary Fig. 10i–k). Consistent with the α-BTX-staining and calcium pulse suppression by the acetylcholine receptor blocker (Fig. 2c, i), NMJ markers *MUSK* and *ACHE* were detected in the muscle and neural clusters (Supplementary Fig. 7d). Together, these results affirm the effectiveness of our induction protocols and highlight their potential to co-induce an even broader range of cell types from bovine ESCs. However, they also present the challenges of precisely controlling cell differentiation processes: variations in protocols, such as 2D versus 3D cultures, can lead to unexpected differences in cell type composition, potentially due to differences in substrate and tissue stiffness, cell density, and other microenvironmental cues that influence cell fate decisions.

## Discussion

In this study, we demonstrated the potential of utilizing bovine ESCs as a stable cell source for cultured beef and steak production. The induction of bovine myocytes and muscle fibers from indefinitely proliferative stem cells provides a scalable and robust foundation for cultured meat manufacturing, particularly given that large-scale expansion of bovine ESCs in bioreactors has already been established^9^. The induction of distinct cell types, such as endothelial cells and neurons, from the same cell source can also simplify the production process. Further protocol refinements may enable the co-induction of additional important cell types, such as fat cells.

All protocols described in this study are serum-free and completed within 15 days, enabling rapid induction advantageous for potential applications. In particular, the 3D protocols yielded higher proportions of skeletal muscle relative to smooth muscle and exhibited high reproducibility. Nevertheless, several practical considerations remain. As the protocols were developed using a single bovine ESC line, evaluation with independent sources of bovine pluripotent stem cells^54^ will be important for reproducibility and scalability. In addition, Matrigel, which is derived from mouse carcinoma cells, should be replaced with animal-free alternatives. Furthermore, the current costs are commercially unfeasible: a 0.1 mm^3^ muscle aggregate derived from 20,000 bovine ESCs costs between 0.39 and 0.99 EUR (Supplementary Data 1), with growth factors, chemical inhibitors, and Matrigel accounting for the primary expenses. While cost optimization is beyond the scope of this study, substantial cost reductions through the mass production of these materials or the development of plant-based alternatives will be essential for potential commercialization.

Moreover, we generated a bovine 3D tissue containing skeletal muscle, neurons, and endothelial cell networks. Several 3D skeletal muscle organoids were previously developed from human pluripotent stem cells, but not from bovine stem cells^28,55–57^. Even though vascularized skeletal muscle tissues were previously engineered, the muscle cells and endothelial cells were separately prepared and assembled^13,57–59^, or the cells were induced through the overexpression of master regulators^60^. Thus, the co-induction of bovine myocytes and endothelial cells without genetic modification as well as the subsequent formation of muscle fibers and endothelial networks within a single tissue represent a crucial step toward the production of structured beef steaks. The stimulation of skeletal muscle by neurons should also facilitate the maturation of the muscle tissue. However, we did not observe a clear positive impact of bovine endothelial networks and neurons on muscle cultures, even after 30 days of culture, and further functionalization and maturation of the blood vessels and early NMJ-like structures will be necessary. As the lumen formation in blood vessels is stimulated by intraluminal flow, the next crucial step should involve establishing the connection between the endothelial networks within the muscle aggregates and in vitro perfusable vasculatures^61–64^. Perfusion through blood vessels is vital for nurturing steak-sized tissues, and perfusion with blood substitutes may improve the flavor of cultured steaks. As the blood vessel formation is also heavily influenced by stroma cells, such as pericytes and fibroblasts, co-inducing bovine stroma cells together with endothelial cells may prove beneficial^26,65,66^. Similarly, further maturation of the early NMJ-like structures may require optimization of the induction protocol and extrinsic signaling cues, as well as the incorporation of additional supporting cell types, such as Schwann cells, during long-term culture^28^.

Despite the remaining challenges, we propose the co-induction of multiple cell types and self-organization of tissues as feasible directions in the production of cultured beef steaks. Our approach will also be complementary to engineering approaches: co-induced cells can be mixed with a bioink and readily used for bioprinting, while muscle aggregates featuring endothelial networks can serve as building blocks for complex tissue assembly. Diverse types of cultured beef will offer novel food options.

## Methods

### Bovine ESC culture

The bovine ESC line was obtained from Dr. Juan Carlos Izpisua Belmonte^8^. The HES7 promoter-luciferase reporter bovine ESC line was described previously^30^. The ESCs were maintained without feeder cells and cultured on Matrigel (Corning, 356231)-coated dishes. Either mTeSR1 medium (STEMCELL Technologies) or StemFit medium (Ajinomoto, StemFit Basic04CT) supplemented with 20 ng/mL bFGF (PeproTech, AF-100-18B), 20 ng/mL Activin A (R&D, 338-AC), and 2.5 μM IWR1 (Tocris, 3532) was used as the maintenance medium. The ESCs were passaged every other day. The cells were trypsinized into single cells by TrypLE solution (Gibco, A1285901) and 0.5 mM ethylenediaminetetraacetic acid (EDTA) in phosphate-buffered saline (PBS) (1:1 mixture) at room temperature for 1 min. Then 2.3 × 10^5^ − 2.8 × 10^5^ cells were seeded on a Matrigel-coated 3.5 cm dish in the maintenance medium containing 5 µM ROCK inhibitor, Y27632 (Sigma, Y0503). The cells were maintained at 37 °C in a humidified atmosphere of 5% CO_2_. The medium was changed the next day into the maintenance medium without Y27632.

### Induction media

#### From day 0 to day 2: PSM medium

The PSM medium is CDMi containing 10 μM CHIR99021 (Sigma, SML1046), 20 ng/ml bFGF, 10 μM SB431542 (Sigma, S4317), and 2 μM DMH1 (Sigma, D8946) as previously described^28,33,67^.

#### From day 2 to day 6: muscle medium_1

The basal medium is DMEM/F12 (Gibco, 11320033) containing 15% Knockout Serum Replacement (Gibco, 10828028), 0.1 mM nonessential amino acids (Gibco, 11140-035), 50 mM 2-mercaptoethanol (Gibco, 31350-010), and 0.5x penicillin/streptomycin (Gibco, 15140-122).

The muscle medium_1 is the basal medium supplemented with 10 ng/ml HGF (R&D, 2207-HG-025/CF), 2 ng/ml IGF (R&D, 791-MG-050), and 20 ng/ml bFGF.

#### From day 2 to day 6: muscle/Endothelial Cells (EC) medium_1

The muscle/EC medium_1 is the basal medium supplemented with 10 ng/ml HGF, 2 ng/ml IGF, 20 ng/ml bFGF, 2 µM forskolin (R&D, 2207-HG-025/CF), and 200 ng/ml VEGF165 (R&D, 2207-HG-025/CF).

#### From day 6 to day 15: muscle medium_2

The muscle medium_2 is the basal medium supplemented with 10 ng/ml HGF and 2 ng/ml IGF.

#### From day 6 to day 15: muscle/EC medium_2

The muscle/EC medium_2 is the basal medium supplemented with 10 ng/ml HGF, 2 ng/ml IGF, and 100 ng/ml VEGF165.

### Bovine muscle induction protocol in 2D (Muscle 2D protocol)

One day before the induction protocol started, 2.3 × 10^4^–2.6 × 10^4^ / cm^2^ bovine ESCs were seeded on Matrigel-coated dishes or plates in the maintenance medium without IWR1 and with 5 μM Y27632. The maintenance medium was changed to the PSM medium the next day (defined as day 0). The cells were incubated in the PSM medium for 2 days, and the medium was changed every day. On day 2, the PSM medium was switched to the muscle medium_1, and the cells were incubated for 4 days with daily medium changes. On day 6, the muscle medium_1 was switched to the muscle medium_2, and the cells were incubated until day 15 with medium changes every other day.

### Bovine muscle and endothelial cell co-induction protocol in 2D (Muscle/EC 2D protocol)

Bovine PSM cells were induced as described above. During days 2–6, the cells were incubated in the muscle/EC medium_1 with daily medium changes. During days 6–15, the cells were incubated in the muscle/EC medium_2 with medium changes every other day.

### Bovine muscle induction protocol in 3D (Muscle 3D protocol)

Bovine PSM cells were induced as described above. On day 2, PSM cells were trypsinized into single cells using TrypLE solution at 37 °C for 3 min. The cells were mechanically dissociated by pipetting and transferred into 1 ml of the muscle medium_1 containing 5 µM Y27632. The cell suspension was centrifuged at 258 × g for 3 min and resuspended into 1 ml of the muscle medium_1 containing Y27632. After one more wash with the muscle medium_1 containing Y27632, the supernatant was completely removed. The cell pellet was resuspended into 600–800 µl of the muscle medium_1 containing Y27632. The cell concentration was adjusted to 133.3 cells/µl with a sufficient amount of the pre-warmed muscle medium_1 containing Y27632. Then 150 µl of the cell suspension (=20,000 cells) was aliquoted into each well of a U-Shaped-Bottom, 96-well-plate (96-well Clear Round Bottom Ultra-Low Attachment Microplate, Corning, 7007) by using a multichannel pipette. The 96-well plate was centrifuged at 258 × g for 2 min for the cells to settle down on the bottom of the plate. On day 3, 100 µl of the medium was carefully removed and replaced with 150 µl of the fresh muscle medium_1 containing 2% Matrigel (growth factor reduced, Corning, 356231). After day 4, the plate was placed on a rotary shaker (Celltron, INFORS HT) with the rotation speed set at 75 r.p.m. The medium was not changed until day 6. On day 6, the aggregates were collected using wide-bore tips and transferred into a 6-well plate containing 3 ml of the muscle medium_2. The aggregates were incubated in the muscle medium_2 until day 15 with medium changes every 3 days.

### Bovine muscle and endothelial cell co-induction protocol in 3D (Muscle/EC 3D protocol)

The aggregate of 20,000 PSM cells was made as described above using the muscle/EC medium_1 instead of the muscle medium_1. During days 6–15, the aggregates were incubated in the muscle/EC medium_2 instead of the muscle medium_2.

### Immunohistochemistry (IHC) for 2D cell cultures

Cells in 2D cultures were washed twice with PBS and fixed in 300 µl of 4% paraformaldehyde (PFA) at room temperature for 15 min. The cells were washed twice with PBSTw (0.2% Tween20 in PBS) for 5 min each, and then treated with the blocking buffer (PBS containing 3% Bovine Serum Albumin (BSA) and 0.2% Tween20) at room temperature for a few hours or at 4 °C overnight. The cells were then incubated with the following primary antibodies in the blocking buffer at 4 °C overnight: anti-TBX6 antibody (1/300 dilution, Abcam, ab38883), anti-SOX2 antibody (1/300 dilution, R&D, MAB2018), anti-BRACHYURY (BRA) antibody (1/300 dilution, R&D, AF2085), anti-SARCOMERIC ALPHA ACTININ (SAA) antibody (1/1000 dilution, Abcam, ab9465), anti-BETA III TUBULIN (TUJ1) antibody (1/500 dilution, Abcam, ab18207), anti- MYOGENIN (MYOG) antibody (1/500 dilution, Abcam, ab124800), anti-VE-CADHERIN (VE-cad) antibody (1/300 dilution, Cell Signaling Technologies, 2500S), anti-NEUROFILAMENT HEAVY POLYPEPTIDE (SMI-32) antibody (1/300 dilution, Abcam, ab8135), anti-MYOSIN HEAVY CHAIN (MHC) antibody (1/300 dilution, R&D, MAB4470), anti-HOXC6 antibody (1/300 dilution, Invitrogen, PA5-41479), anti-ALPHA-SMOOTH MUSCLE ACTIN (α-SMA) antibody (1/500 dilution, Sigma, A5228), anti-NEUROFILAMENT (NEFM) antibody (1/500 dilution, MilliporeSigma, MAB5254), anti-DESMIN antibody (1/500 dilution, Abcam, ab15200), anti-NG2 antibody (1/300 dilution, Invitrogen, MA5-24247), or α-BUNGAROTOXIN (α-BTX) Conjugates-594nm (1/500 dilution, Invitrogen, B13423). The next day, the cells were washed 3 times with PBSTw for 15 min each. Then the cells were incubated with the following secondary antibodies at a 1/500 dilution together with DAPI (1/500 dilution, Invitrogen, 62247) in PBSTw at 4 °C overnight: Alexa Fluor 594 Goat anti-Rabbit IgG (H + L) (Invitrogen, A-11037), Alexa Fluor 647 Donkey anti-Goat IgG (H + L) (Invitrogen, A32849), or Alexa Fluor 488 Goat anti-Mouse IgG (H + L) (Invitrogen, A-11029). The cells were washed 3 times with PBSTw for 15 min each, and images were taken with an FV3000 confocal microscope (Olympus, FV3000 Fluoview RS software) or LSM980 (Zeiss, ZEN). Tiled images were stitched together using ImageJ Grid/Collection stitching. The fluorescent signal-positive area was measured using ImageJ thresholding.

### IHC for 3D aggregates

Aggregates in 3D cultures were collected using wide-bore tips into 2 ml Eppendorf tubes. After 2 washes with PBS, the aggregates were fixed in 500 μl of 4% PFA at 4 °C overnight. They were washed 3 times with PBSTt (0.5% Triton X-100 in PBS) for 15 min each, and treated with the blocking buffer (PBS containing 5% Normal Goat Serum and 0.5% Triton X-100) at 4 °C overnight on a shaker. Then the aggregates were incubated with the primary antibodies described above in the blocking buffer at 4 °C for 2 days on a shaker. They were washed 3 times with PBSTt for 20 min each and incubated with the secondary antibodies described above in PBSTt at 4 °C overnight on a shaker. After 3 washes with PBSTt for 20 min each, the aggregates underwent a clearing treatment. They were washed once with MilliQ and then embedded in 1% Agarose (low gelling temperature, Sigma, A9414) using a handmade rectangular box. The aggregates were then incubated with 100% Methanol in a glass bottle at room temperature for 2 days in the dark. The Methanol was changed daily. Then the Methanol was replaced with BABB (a mixture of Benzyl benzoate and Benzyl alcohol at a 1:1 ratio), and the aggregates were incubated at room temperature for 2 days in the dark. The BABB solution was changed daily. Images were taken using a MuVi-SPIM Light-sheet microscope with a clear chamber and BABB (Z = 2 μm, XY = 0.65 μm) or Lightsheet 7 (Zeiss) with fructose-glycerol solution (Z = 2 μm). Tiled images were stitched together using the MuVi-SPIM Light-sheet microscope’s software. The volume of the fluorescent signal-positive region was measured using the ilastik software with a pixel configuration and a custom-written Python script. Once the masked images of Z-stacks were generated by ilastik, the positive pixel values within each mask were counted, and these pixel counts were converted into a volume.

### Time-lapse imaging and quantification of HES7 oscillation

The HES7 promoter-luciferase reporter cells were incubated in the PSM medium containing 0.5 mM luciferin. The medium was changed daily. The luciferase activity was recorded using a Kronos Dio Luminometer (Atto, Kronos control software v2.3) every 10 min with 1 min exposure. The detrended signal was calculated by pyBOAT^68^.

### Calcium oscillation measurement

Calcium activity was visualized with Fluo-8 AM (Abcam, ab112129) following the manufacturer’s protocol. Briefly, day-15 or day-18 cultured cells were incubated with a mixture of Component A, B, and C at 37 °C for 30 min. The Fluo-8 signals were recorded using the Thunder Imager Live Cell & 3D assay (Leica, LAS X software) or Eclipse Ti2 inverted motorized stand with hardware autofocus (Nikon, NIS-Elements software). Images were collected every 100 ms for 2 min prior to treatment. Cells were then incubated with 10 µM tubocurarine chloride pentahydrate (curare; Sigma-Aldrich, T2379) for 30 min, after which Fluo-8 signals were recorded again under the same imaging conditions. For analyses, the Fluo-8 intensity from a randomly selected cell was measured using ImageJ. The signal was denoised using the Savitzky-Golay filter with a 50 window size in Python. The amplitudes between a signal peak and its adjacent two valleys were measured, and the peak was counted only if the smaller amplitude was larger than half of the larger amplitude. The total number of oscillation peaks in 2 min was manually counted.

### Quantitative RT-PCR (qRT-PCR)

Total RNA was extracted with RNeasy kit (Qiagen, 74104), and 1 µg RNA was reverse-transcribed with QuantiTect Reverse Transcription kit (Qiagen, 205311) to generate cDNA. The cDNA level was measured by a LightCycler 480 II (Roche) with LightCycler 480 SYBR Green I Master (Roche, 4707516001). The primer sequences are listed in Supplementary Data 2. The expression levels of the target genes were normalized by the *GAPDH* level.

### α-BTX quantification

α-BTX signals were analyzed with the batch mode of Neuromuscular Junction (NMJ) Analysis Pipeline (https://github.com/YuyaSanaki/NMJ-analysis). Muscle and neuron channels were segmented independently from raw fluorescence using Otsu’s method. Euclidean distance transforms (EDTs) were computed on each mask background and converted to micrometers using image metadata pixel size. α-BTX signals were processed by a wide-Gaussian haze subtraction, then normalized to the 99.9th percentile of haze-subtracted intensity. α-BTX spots were detected with a difference-of-Gaussians filter over a 5–12 µm diameter range. Detection sensitivity was set by the default automatic threshold (conservative). Based on their shortest Euclidean distance to the muscle and neuron masks, each detected spot was assigned to one of four classes. Early-NMJ-like: within 1.0 µm of both muscle and neuron masks. Muscle-associated: within 1.0 µm of the muscle mask only. Neuron-associated: within 1.0 µm of the neuron mask only. Orphaned: farther than 1.0 µm from both muscle and neuron masks.

### Endothelial network connectivity analysis

Endothelial network connectivity was analyzed with VascuMap software using a UNet architecture with a mixed vision transformer encoder^69^. The model, trained on endothelial cell images co-cultured with bovine muscle, labeled manually on an iPad 11 Pro with Photoshop and binarized via Python script. Images were divided into training and validation sets (70%/30%) with a fixed seed of 42. Training involved 200 epochs, batch size 16, RAdam optimizer, initial learning rate 0.001, step scheduler for rate reduction, and augmentations like flips and rotations. The loss function combined binary cross-entropy and dice coefficient, with the best IoU metric model on the validation set retained. For unseen data, VascuMap’s probability map was binarized using hysteresis thresholding (thresholds: 0.15, 0.5). The characterization of the binary masks returned the vessel area, total number of vessel segments, total number of branching points, and mean number of vessel segments per branch point.

### Single-cell RNA sequencing (scRNA-seq)

#### Cell preparation for 2D cultures

Cells were induced with individual protocols (Muscle 2D or Muscle/EC 2D) for 15 days using a Matrigel-coated 12-well plate. After 2 washes with PBS, the samples were trypsinized into single cells through the treatment with the TrypLE solution and 0.5 mM EDTA in PBS (1:1 mixture) at 37 °C for 10 min. The cells were mechanically dissociated by pipetting and transferred into PBS containing 0.1% bovine serum albumin (BSA). The cell suspension was centrifuged at 160 × g for 3 min and resuspended into 1 ml of 0.1% BSA in PBS. After one more wash with 0.1% BSA in PBS, the supernatant was completely removed. The cell pellet was resuspended in Cell Prep Buffer (PBS with 0.1% poly(vinyl alcohol) (PVA) and 1 mM EDTA) and then filtered through a 35-µm cell strainer.

#### Cell preparation for 3D aggregates

3D aggregates were induced with individual protocols (Muscle 3D or Muscle/EC 3D) for 15 days. 30 aggregates were collected with wide-bore tips into 2 ml Eppendorf tubes. After 2 washes with PBS, the samples were trypsinized into single cells through the treatment with 0.25 mM Trypsin-EDTA at 37 °C for 10 min. The cells were mechanically dissociated by pipetting and transferred into of 0.1% BSA in PBS. The dissociated cells were washed twice with 0.1% BSA in PBS and resuspended in Cell Prep Buffer and then filtered through a 35-µm cell strainer.

#### Barcoding and sequencing

Transcripts of individual cells were barcoded, and dual-indexed cDNA libraries were generated with the Chromium Single Cell G Chip Kit and Chromium Single Cell 3′ GEM, Library & Gel Bead Kit v3.1 (10x Genomics) by using the Chromium Controller (10x Genomics, firmware version 4.00) according to the manufacturer’s protocol. The finished cDNA libraries were sequenced with NextSeq2000 (Illumina). 28, 10, 10, and 90 base pairs were read for 10x barcodes and unique molecular identifiers (UMIs), for the i7 index, for the i5 index, and for fragmented cDNA, respectively.

#### Read alignment

Sequenced reads were aligned to the bovine genome (ARS-UCD1.2 Ensembl release 110) and counted to generate the feature-barcode matrices with the CellRanger pipeline (version 7.0.1, 10x Genomics). The reference index for the pipeline was prepared with the cellranger mkref command in the same CellRanger package, based on the aforementioned genome reference and the gene annotation file from ARS-UCD1.2 Ensembl release 106. The reads containing the same UMIs were collapsed as a single count. The basic statistics of sequencing results are shown in Supplementary Data 3.

Using R version 4.3.1 and Seurat version 5.1.0, the single-cell gene expression matrices underwent quality control based on the following criteria: (1) the number of detected genes exceeded 1500 across all 4 samples; (2) the total number of detected RNA molecules was greater than 50,000 in the Muscle 2D sample and greater than 40,000 in the other samples; and (3) the proportion of detected mitochondrial genes was less than 31% in the Muscle/EC 2D sample, less than 32% in the Muscle/EC 3D sample, and less than 33% in the Muscle 2D and Muscle 3D samples.

The gene expression matrices from the 4 samples corresponding to individual protocols were integrated into a single Seurat object using sctransform normalization, implemented in Seurat. For dimensionality reduction and clustering, the integrated dataset was analyzed using the fastMNN method, considering the first 20 principal components (PCs) from PCA. Clustering was performed at a resolution of 0.003, identifying muscle, neural, and endothelial cell clusters. Subsequently, the muscle and neural cell clusters were extracted into separate Seurat objects. Each subset was further analyzed by applying fastMNN for dimensionality reduction and clustering, with subclusters generated at resolutions of 0.01 for muscle cells and 0.03 for neural cells.

### Sample definition

All measurements were taken from distinct experiments (biological replicates).

### Reporting summary

Further information on research design is available in the Nature Portfolio Reporting Summary linked to this article.

## Supplementary information

## Source data

## Data Availability

Source data are provided with this paper. The scRNA-seq data generated in this study have been deposited in the ArrayExpress database under accession code #E-MTAB-14714. Source data are provided with this paper.

## Code availability

The custom scripts used are available from Github [https://git.embl.de/grp-mif/image-analysis/meatball_analysis], [https://github.com/LRpz/VascuMap], and [https://github.com/YuyaSanaki/NMJ-analysis].
