# Supplementary material for: Self-organization of vascularized muscle from bovine embryonic stem cells
Source: Nat Commun. 2026 Sep 2;17:8975. doi: 10.1038/s41467-026-76569-2 (PMC13538390; doi:10.1038/s41467-026-76569-2)
Supplement: Supplementary file 1 [file 41467_2026_76569_MOESM12_ESM.pdf]

nature portfolio

Corresponding author(s): Miki Ebisuya
Marina Sanaki-Matsumiya

Last updated by author(s): Feb 12, 2026

Reporting Summary

Nature Portfolio wishes to improve the reproducibility of the work that we publish. This form provides structure for consistency and transparency in reporting. For further information on Nature Portfolio policies, see our Editorial Policies and the Editorial Policy Checklist.

Statistics

For all statistical analyses, confirm that the following items are present in the figure legend, table legend, main text, or Methods section.

n/a	Confirmed	
☐	☒	The exact sample size (*n*) for each experimental group/condition, given as a discrete number and unit of measurement
☐	☒	A statement on whether measurements were taken from distinct samples or whether the same sample was measured repeatedly
☐	☒	The statistical test(s) used AND whether they are one- or two-sided<br>*Only common tests should be described solely by name; describe more complex techniques in the Methods section.*
☒	☐	A description of all covariates tested
☒	☐	A description of any assumptions or corrections, such as tests of normality and adjustment for multiple comparisons
☐	☒	A full description of the statistical parameters including central tendency (e.g. means) or other basic estimates (e.g. regression coefficient) AND variation (e.g. standard deviation) or associated estimates of uncertainty (e.g. confidence intervals)
☐	☒	For null hypothesis testing, the test statistic (e.g. *F*, *t*, *r*) with confidence intervals, effect sizes, degrees of freedom and *P* value noted<br>*Give P values as exact values whenever suitable.*
☒	☐	For Bayesian analysis, information on the choice of priors and Markov chain Monte Carlo settings
☒	☐	For hierarchical and complex designs, identification of the appropriate level for tests and full reporting of outcomes
☒	☐	Estimates of effect sizes (e.g. Cohen's *d*, Pearson's *r*), indicating how they were calculated

Our web collection on statistics for biologists contains articles on many of the points above.

Software and code

Policy information about availability of computer code

Data collection	The following softwares were used: Olympus FV3000 Fluoview RS software for data collection by FV3000 confocal microscopy, Harmony software v4.9 for data collection by Opera Phenix HSC, Luxendo processor software v3.0 for data collection by MuViSpim CS, Kronos control software v2.3 for data collection by Atto, Olympus cellSens software for data collection by IX73 Inverted Microscope, LAS X software for data collection by Thunder Imager Live Cell & 3D assay, NIS-Elements software v5.42.05 for data collection by NIKON TIRF FRAP (Super-resolution Microscopes, H5), and ZEN software for data collection by Airyscan2 LSM980 and ZEISS LIGHTSHEET Z.1.
Data analysis	python_3.8, Fiji_1.53f51, R_4.3.1, Platform: x86_64-apple-darwin20 (64-bit), tidyverse 2.0.0, Seurat_5.1.0, SeuratWrappers_0.3.4, monocle3_1.4.11, SingleCellExperiment_1.24.0, ggthemes_5.1.0 , gridExtra_2.3 enrichR_3.2, org.Bt.eg.db_3.18.0, AnnotationDbi_1.64.1, clusterProfiler_4.10.1, torch_1.12.1, albumentations_0.4.3, segmentation_models_pytorch_0.3.0, catalyst_20.12, networkx_2.8.4, sknw_0.1, alphashape_1.3.1, scikit-image_0.19.2, 0.26.0, ComponentVersionPython_3.12.13, numpy_2.5.0, 2.3.5, scipy_1.18.0, 1.16.3, pandas_3.0.3, 2.3.3, matplotlib_3.11.0, seaborn_0.13.2, streamlit_1.58.0, aicspylibczi_3.3.1, nd_20.11.3, readlif_0.6.6, oirfile_2026.4.25, tifffile_2026.6.1<br>The custom scripts used are available from Github [https://git.embl.de/grp-mif/image-analysis/meatball_analysis], [https://github.com/LRpz/VascuMap], and [https://github.com/YuyaSanaki/NMJ-analysis].

For manuscripts utilizing custom algorithms or software that are central to the research but not yet described in published literature, software must be made available to editors and reviewers. We strongly encourage code deposition in a community repository (e.g. GitHub). See the Nature Portfolio guidelines for submitting code & software for further information.

Data

Policy information about availability of data

All manuscripts must include a data availability statement. This statement should provide the following information, where applicable:

- Accession codes, unique identifiers, or web links for publicly available datasets
- A description of any restrictions on data availability
- For clinical datasets or third party data, please ensure that the statement adheres to our policy

The scRNA-seq data are available at https://www.ebi.ac.uk/biostudies/arrayexpress/studies/E-MTAB-14714. All data are available within the Article, Supplementary Information, or Source Data file.

Research involving human participants, their data, or biological material

Policy information about studies with human participants or human data. See also policy information about sex, gender (identity/presentation), and sexual orientation and race, ethnicity and racism.

Reporting on sex and gender	The sex of the bovine ESCs was not considered, as this study is a proof of principle aimed at demonstrating the self-organization ability of bovine ESCs rather than evaluating the efficiency across different cell lines and sexes.
Reporting on race, ethnicity, or other socially relevant groupings	*Please specify the socially constructed or socially relevant categorization variable(s) used in your manuscript and explain why they were used. Please note that such variables should not be used as proxies for other socially constructed/relevant variables (for example, race or ethnicity should not be used as a proxy for socioeconomic status).*<br>*Provide clear definitions of the relevant terms used, how they were provided (by the participants/respondents, the researchers, or third parties), and the method(s) used to classify people into the different categories (e.g. self-report, census or administrative data, social media data, etc.)*<br>*Please provide details about how you controlled for confounding variables in your analyses.*
Population characteristics	*Describe the covariate-relevant population characteristics of the human research participants (e.g. age, genotypic information, past and current diagnosis and treatment categories). If you filled out the behavioural & social sciences study design questions and have nothing to add here, write "See above."*
Recruitment	*Describe how participants were recruited. Outline any potential self-selection bias or other biases that may be present and how these are likely to impact results.*
Ethics oversight	*Identify the organization(s) that approved the study protocol.*

Note that full information on the approval of the study protocol must also be provided in the manuscript.

Field-specific reporting

Please select the one below that is the best fit for your research. If you are not sure, read the appropriate sections before making your selection.

☒ Life sciences ☐ Behavioural & social sciences ☐ Ecological, evolutionary & environmental sciences

For a reference copy of the document with all sections, see nature.com/documents/nr-reporting-summary-flat.pdf

Life sciences study design

All studies must disclose on these points even when the disclosure is negative.

Sample size	No statistical method was used to predetermine the sample size. The sample size was empirically determined based on our previous work using the same cell line and a similar protocol (Lazaro et all, Cell Stem Cell, 2023). Actual sample sizes are provided in the figure legends.
Data exclusions	No data was excluded.
Replication	All attempts of replication have been successful. The variability was reported in the graphs and figure legends.
Randomization	Randomization was not used in this study, as a single researcher conducted the majority of the experiments.
Blinding	Blinding was not used in this study, as a single researcher conducted the majority of the experiments. Data analyses are semi-automated and follow clear criteria.

Reporting for specific materials, systems and methods

We require information from authors about some types of materials, experimental systems and methods used in many studies. Here, indicate whether each material, system or method listed is relevant to your study. If you are not sure if a list item applies to your research, read the appropriate section before selecting a response.

Materials & experimental systems

n/a	Involved in the study
☐	☒ Antibodies
☐	☒ Eukaryotic cell lines
☒	☐ Palaeontology and archaeology
☒	☐ Animals and other organisms
☒	☐ Clinical data
☒	☐ Dual use research of concern
☒	☐ Plants

Methods

n/a	Involved in the study
☒	☐ ChIP-seq
☒	☐ Flow cytometry
☒	☐ MRI-based neuroimaging

Antibodies

Antibodies used

All antibodies used in this study are commercially available.

Primary antibody
anti-TBX6 antibody (1/300 dilution, Abcam, ab38883), anti-SOX2 antibody (1/300 dilution, R&D, MAB2018), anti-BRACHYURY antibody (1/300 dilution, R&D, AF2085), anti-SARCOMERIC ALPHA ACTININ (SAA) antibody (1/1000 dilution, Abcam, ab9465), anti-BETA III TUBULIN (TUJ1) antibody (1/500 dilution, Abcam, ab18207), anti-MYOGENIN antibody (1/1000 dilution, Abcam, ab124800), anti-VE-CADHERIN antibody (1/300 dilution, Cell Signaling Technologies, 2500S), anti-NEUROFILAMENT HEAVY POLYPEPTIDE (SMI-32) antibody (1/300 dilution, Abcam, ab8135), anti-MYOSIN HEAVY CHAIN (MHC) antibody (1/300 dilution, R&D, MAB4470), anti-NEUROFILAMENT (NEFM) antibody (1/500 dilution, Sigma, MAB5254), anti-ALPHA-SMOOTH MUSCLE ACTIN (α-SMA) antibody (1/500 dilution, Sigma, A5228), anti-DESMIN antibody (1/300 dilution, Abcam, ab15200), anti-NG2 antibody (1/300 dilution, Invitrogen, MA5-24247), or anti-HOXC6 antibody (1/300 dilution, Invitrogen, PA5-41479)

Secondary antibody
DAPI (1/500 dilution, Invitrogen, 62247), Alexa Fluor™ Plus 594 Goat anti-Rabbit IgG (H+L) (1/500 dilution, Invitrogen, A-11037), Alexa Fluor™ Plus 647 Donkey anti-Goat IgG (H+L) (1/500 dilution, Invitrogen, A32849), Alexa Fluor™ Plus 488 Goat anti-Mouse IgG (H+L) (1/500 dilution, Invitrogen, A-11029), Alexa Fluor™ Plus 647 Goat anti-Rat IgG (H+L) (1/500 dilution, Invitrogen, A-48265) or Alpha-Bungarotoxin Conjugates-594nm (1/500 dilution, Invitrogen, B13423)

Validation

anti-TBX6 antibody (Abcam, ab38883), anti-SOX2 antibody (R&D, MAB2018), anti-BRACHYURY antibody (R&D, AF2085), anti-SARCOMERIC ALPHA ACTININ (SAA) antibody (Abcam, ab9465), anti-BETA III TUBULIN (TUJ1) antibody (Abcam, ab18207), anti-MYOGENIN antibody (Abcam, ab124800), anti-VE-CADHERIN antibody (Cell Signaling Technologies, 2500S), anti-NEUROFILAMENT HEAVY POLYPEPTIDE (SMI-32) antibody (Abcam, ab8135), anti-MYOSIN HEAVY CHAIN (MHC) antibody (R&D, MAB4470), anti-NEUROFILAMENT (NEFM) antibody (Sigma, MAB5254), anti-ALPHA-SMOOTH MUSCLE ACTIN (α-SMA) antibody (Sigma, A5228), anti-DESMIN antibody (Abcam, ab15200), anti-NG2 antibody (Invitrogen, MA5-24247), anti-HOXC6 antibody (Invitrogen, PA5-41479), DAPI (Invitrogen, 62247), Alexa Fluor™ Plus 594 Goat anti-Rabbit IgG (H+L) (Invitrogen, A-11037), Alexa Fluor™ Plus 647 Donkey anti-Goat IgG (H+L) (Invitrogen, A32849), Alexa Fluor™ Plus 488 Goat anti-Mouse IgG (H+L) (Invitrogen, A-11029), Alexa Fluor™ Plus 647 Goat anti-Rat IgG (H+L) (Invitrogen, A-48265) or Alpha-Bungarotoxin Conjugates-594nm (Invitrogen, B13423)

Eukaryotic cell lines

Policy information about cell lines and Sex and Gender in Research

Cell line source(s)

The bovine ESC line (#A) was obtained from Dr. Juan Carlos Izpisua Belmonte.

Authentication

The cell line was not authenticated.

Mycoplasma contamination

Cells were regularly tested and reported negative for mycoplasma contamination.

Commonly misidentified lines
(See ICLAC register)

NA

Plants

Seed stocks

Report on the source of all seed stocks or other plant material used. If applicable, state the seed stock centre and catalogue number. If plant specimens were collected from the field, describe the collection location, date and sampling procedures.

Novel plant genotypes

Describe the methods by which all novel plant genotypes were produced. This includes those generated by transgenic approaches, gene editing, chemical/radiation-based mutagenesis and hybridization. For transgenic lines, describe the transformation method, the number of independent lines analyzed and the generation upon which experiments were performed. For gene-edited lines, describe the editor used, the endogenous sequence targeted for editing, the targeting guide RNA sequence (if applicable) and how the editor was applied.

Authentication

Describe any authentication procedures for each seed stock used or novel genotype generated. Describe any experiments used to assess the effect of a mutation and, where applicable, how potential secondary effects (e.g. second site T-DNA insertions, mosiacism, off-target gene editing) were examined.
