# Supplementary material for: Self-organization of vascularized muscle from bovine embryonic stem cells
Source: Nat Commun. 2026 Sep 2;17:8975. doi: 10.1038/s41467-026-76569-2 (PMC13538390; doi:10.1038/s41467-026-76569-2)
Supplement: Supplementary file 2 [file 41467_2026_76569_MOESM1_ESM.pdf]

Supplementary Information for

Self-organization of vascularized muscle from bovine embryonic stem cells

Marina Sanaki-Matsumiya[1,2*], Yuya Sanaki[2], Casandra Villava[1], Luca Rappez[1], Nicola Gritti[1], Fumio Nakaki[1], James Sharpe[1], Kristina Haase[1], Jun Wu[3,4,5], Miki Ebisuya[1,6*]

[1]European Molecular Biology Laboratory (EMBL) Barcelona, Dr. Aiguader 88, 08003 Barcelona, Spain.

[2]Life Science Center for Survival Dynamics, Tsukuba Advanced Research Alliance (TARA), University of Tsukuba, 1-1-1 Tennodai, Tsukuba, Ibaraki 305-8577, Japan.

[3]Department of Molecular Biology, University of Texas Southwestern Medical Center, Dallas, TX 75390-9148, USA.

[4]Hamon Center for Regenerative Science and Medicine, University of Texas Southwestern Medical Center, Dallas, TX 75390-9148, USA.

[5]Cecil H. and Ida Green Center for Reproductive Biology Sciences, University of Texas Southwestern Medical Center, Dallas, TX 75390, USA

[6]Cluster of Excellence Physics of Life, TU Dresden, Dresden, Germany.

* Correspondence to M Sanaki-Matsumiya (marina.sanaki.matsumiya@tara.tsukuba.ac.jp) or M Ebisuya (miki.ebisuya@tu-dresden.de)

- Supplementary Figures 1-10

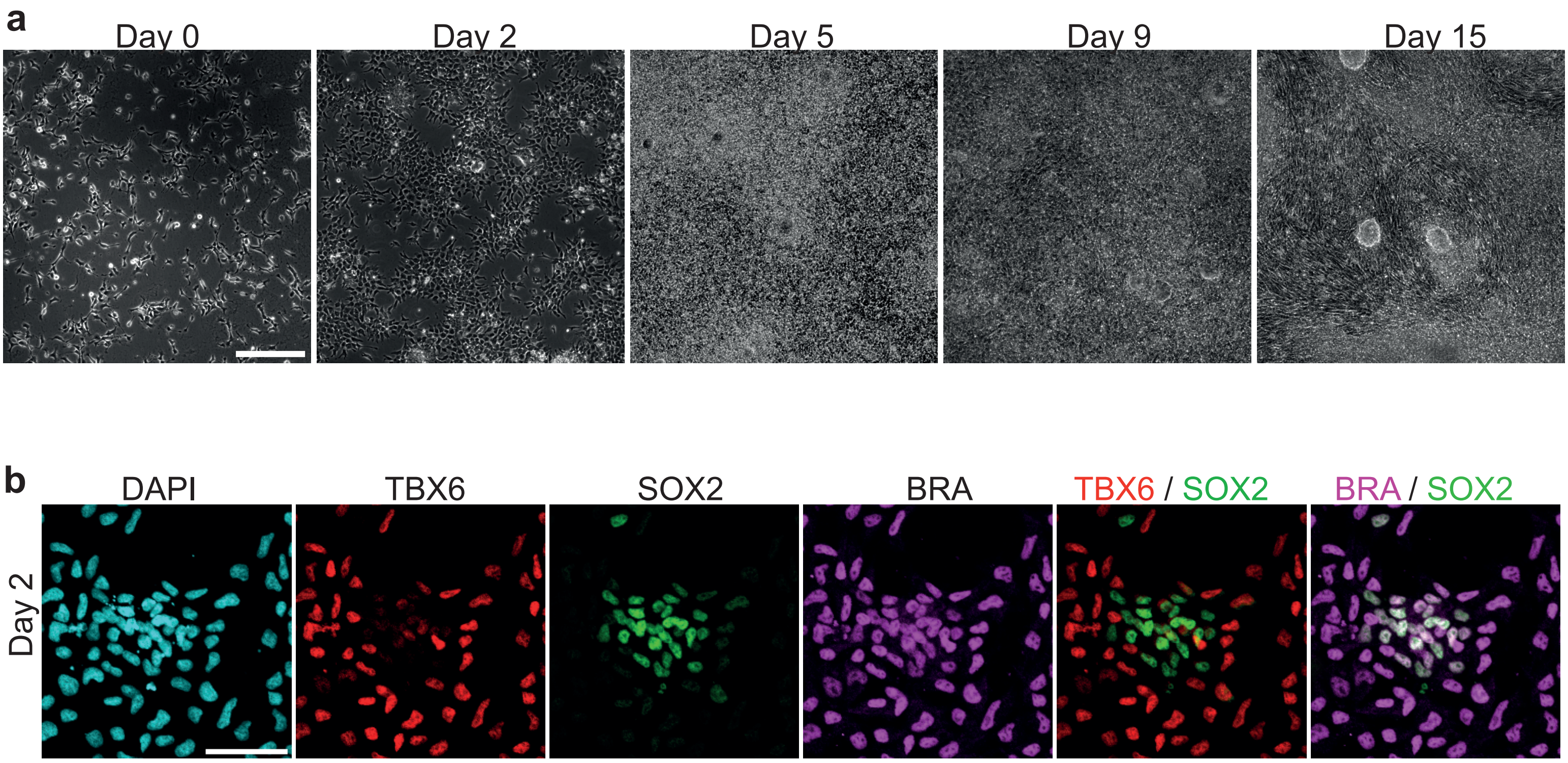

Supplementary Figure 1 Induction of PSM and muscle fibers from bovine ESCs
a, Bright field images over the time course of the muscle 2D protocol. The enlarged views of these images are shown in Fig. 1a. **b,** IHC images of the day-2 culture. The enlarged views of Fig. 1b.
Scale bars: 300 μm (a) and 50 μm (b).

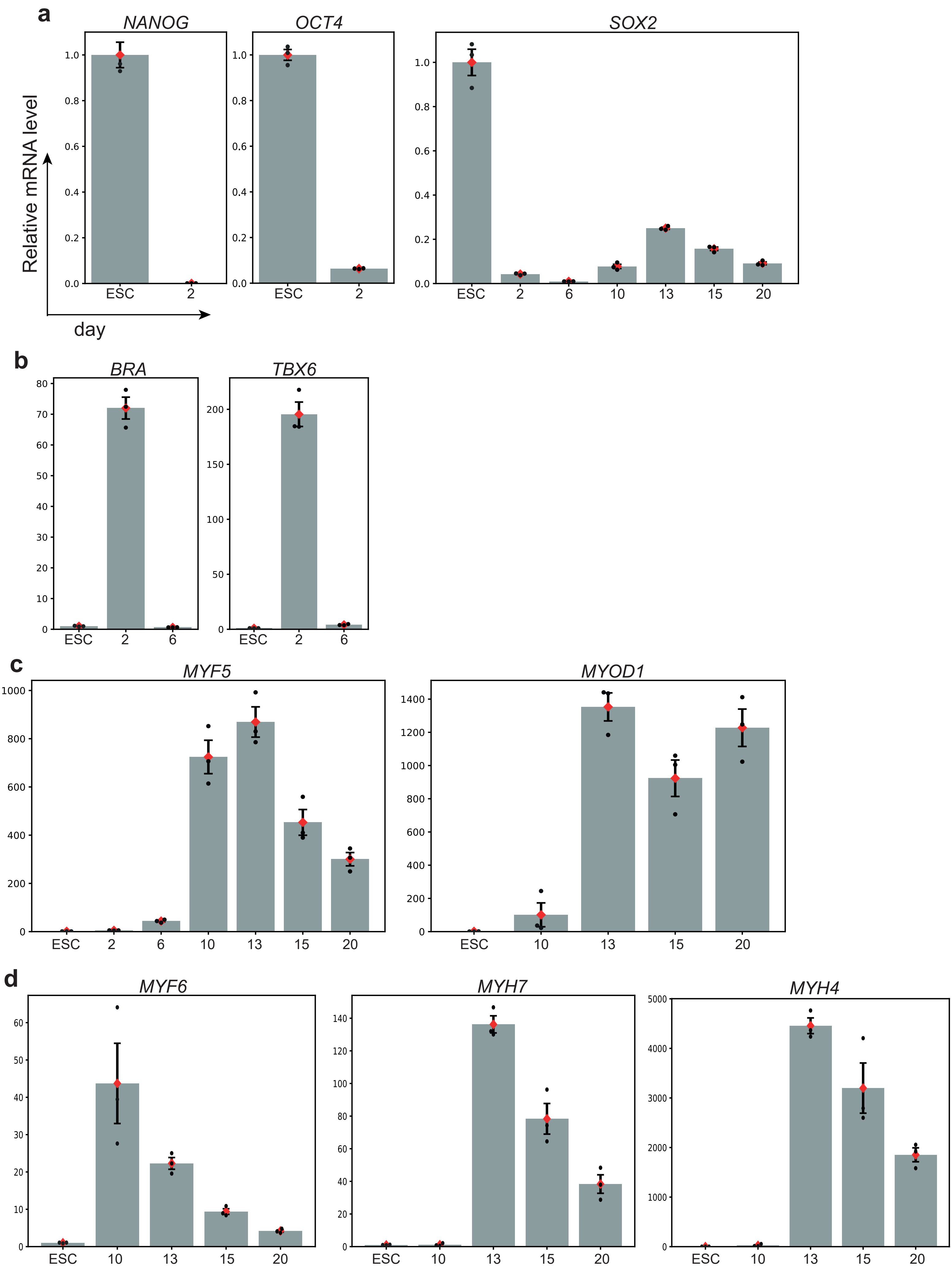

a
NANOG
OCT4
SOX2
Relative mRNA level
day
ESC
2
6
10
13
15
20
b
BRA
TBX6
c
MYF5
MYOD1
d
MYF6
MYH7
MYH4

Supplementary Figure 2 qRT-PCR measurements

Relative mRNA levels of selected marker genes in bovine ESCs and the time course of the muscle 2D protocol. The values were normalized to *GAPDH* expression, and the average values of ESCs were set to 1. Mean ± SEM. N = 3 dishes from 3 independent experiments. **a,** Pluripotency markers. **b,** PSM markers. **c,** Myoblast markers. **d,** Myocyte markers. Primer information is in Supplementary Table 2.
Source data are provided as a Source Data file.

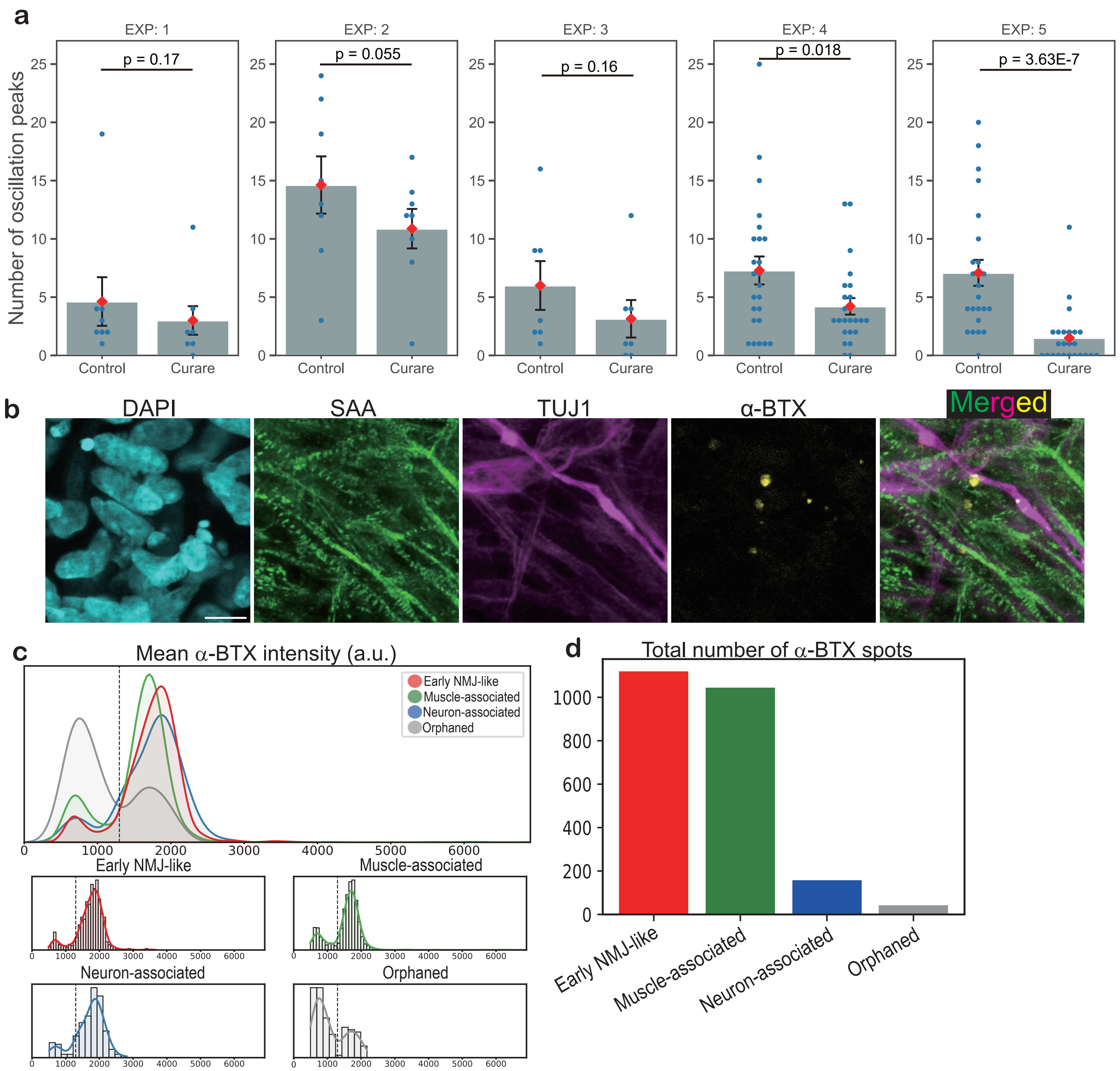

Supplementary Figure 3 Co-induction of muscle and neural cells

a, Quantification of the number of calcium oscillation peaks within 2 min in the absence and presence of curare. Results of 5 independent experiments. Mean ± SEM. P-values are from two-sided paired t-test. The summed data are shown in Fig. 2c. **b,** IHC images of the day-15 cultures with the NMJ marker α-BTX. Scale bar: 10 μm. **c,** α-BTX signal intensity. α-BTX-reactive spots were classified based on their proximity to muscle fibers and neurons. Early NMJ-like: within 1 μm of both muscle fibers and neurons. Muscle-associated: within 1 μm of muscle fibers but not neurons. Neuron-associated: within 1 μm of neurons but not muscle fibers. Orphaned: farther than 1 μm from both muscle fibers and neurons. Mean α-BTX intensity was quantified for N = 1268 (early NMJ-like), 1319 (muscle-associated), 183 (neuron-associated), and 129 (orphaned) spots from 67 images across 20 independent experiments. As the intensity distribution was bimodal, only spots with intensities above the threshold (1298) were included in subsequent analyses. **d,** Number of α-BTX-reactive spots with intensities above the threshold in c. Source data are provided as a Source Data file.

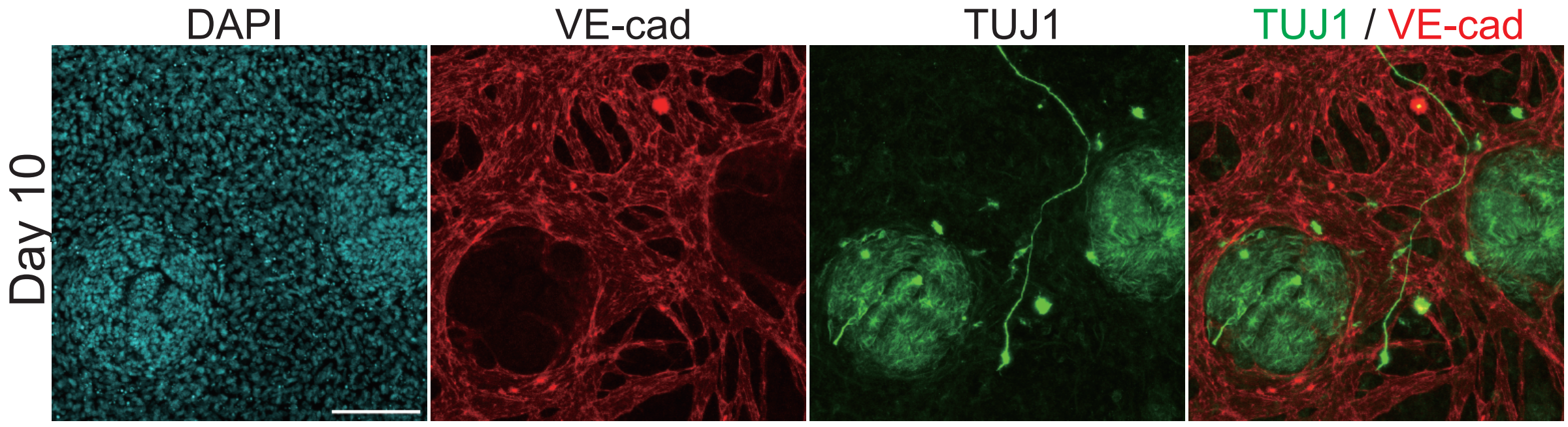

Supplementary Figure 4 Co-induction of endothelial and neural cells from bovine ESCs
IHC images of the day-10 culture of the muscle/EC 2D protocol. TUJ1 and VE-cad are neuronal and endothelial cell markers, respectively. 3 independent experiments showed similar staining patterns. Scale bar: 150 µm.

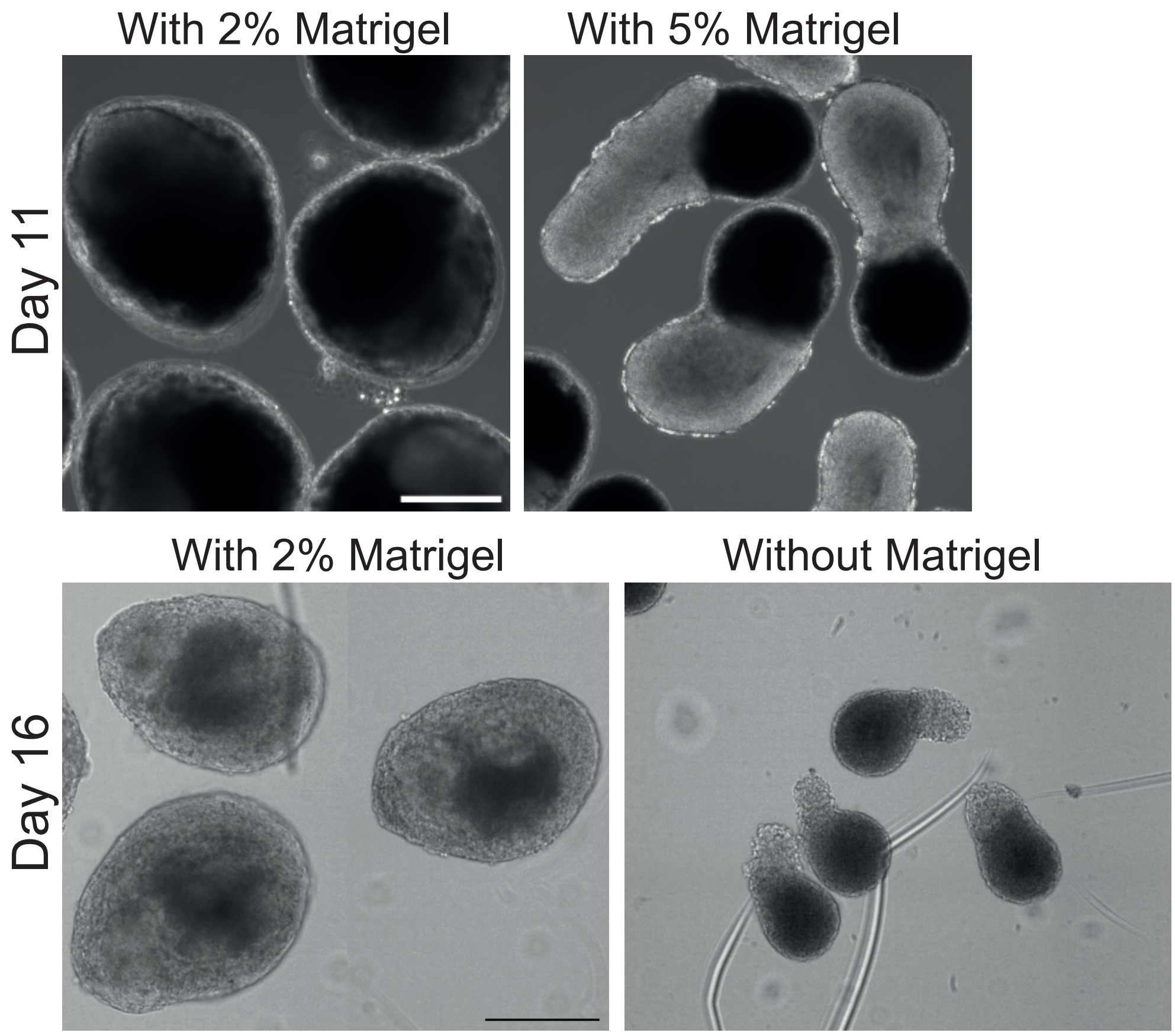

Supplementary Figure 5 Effects of Matrigel on aggregates

Bright field images of the muscle aggregates made without or with (2% or 5%) Matrigel in the medium. 2% is the standard concentration. 3 independent experiments showed similar patterns. Scale bars: 300 μm.

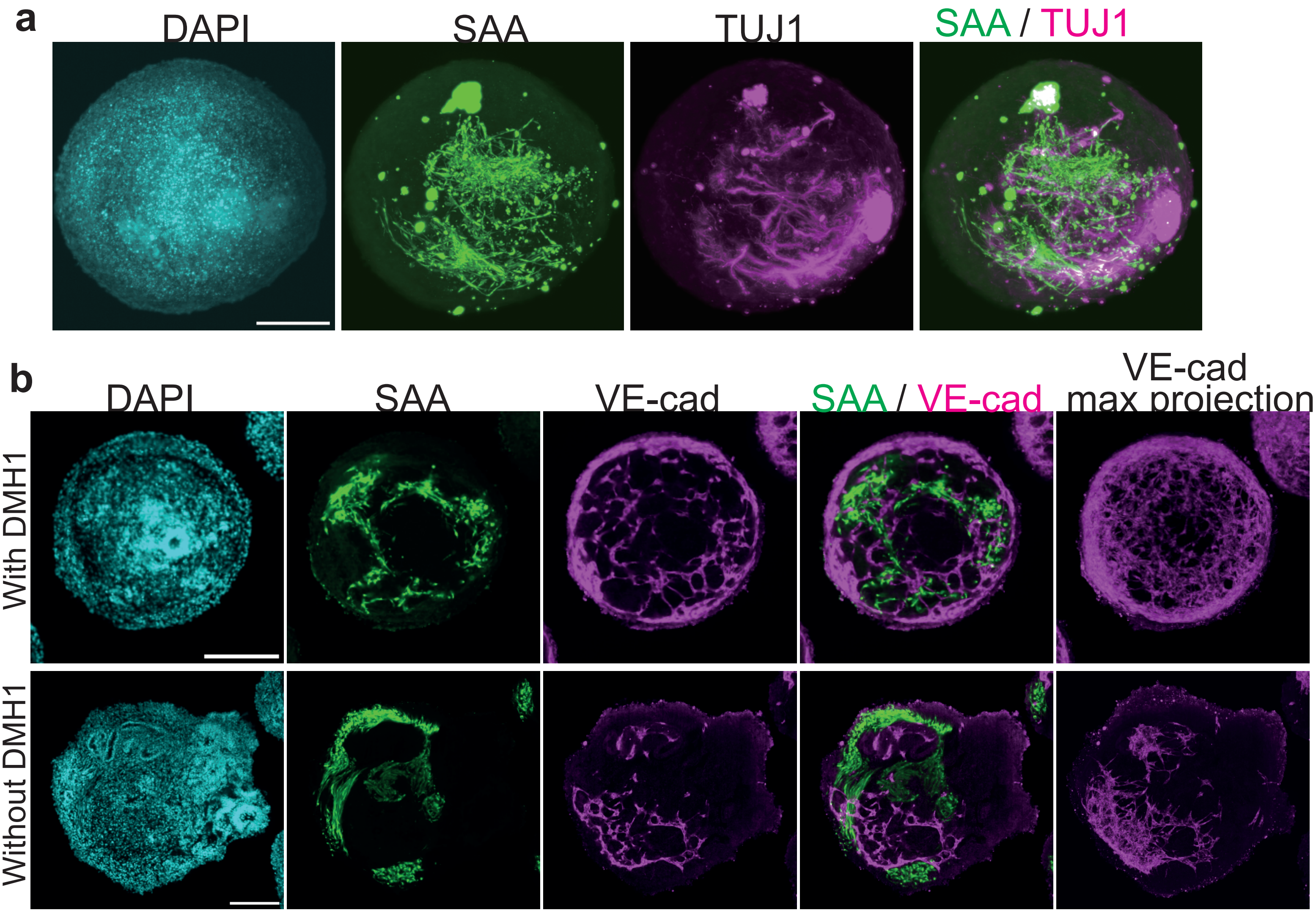

Supplementary Figure 6 Co-induction of muscle, neural, and endothelial cells in 3D
a, IHC images of the day-15 aggregate of the muscle/EC 3D protocol. SAA and TUJ1 are muscle fiber and neuronal markers, respectively. 8 independent experiments showed similar staining patterns. The max projection was shown. **b,** IHC images of the day-15 aggregates. The aggregate made with the standard PSM medium with DMH1 (the BMP inhibitor) showed a uniform VE-cad-positive endothelial network throughout the tissue (top), while the one made without DMH1 showed local endothelial domains (bottom). The max projection of VE-cad used the Z-stacks within the 40 μm range around the central slice. 5-15 independent experiments showed similar patterns. Scale bars: 150 μm (a) and 200 μm (b).

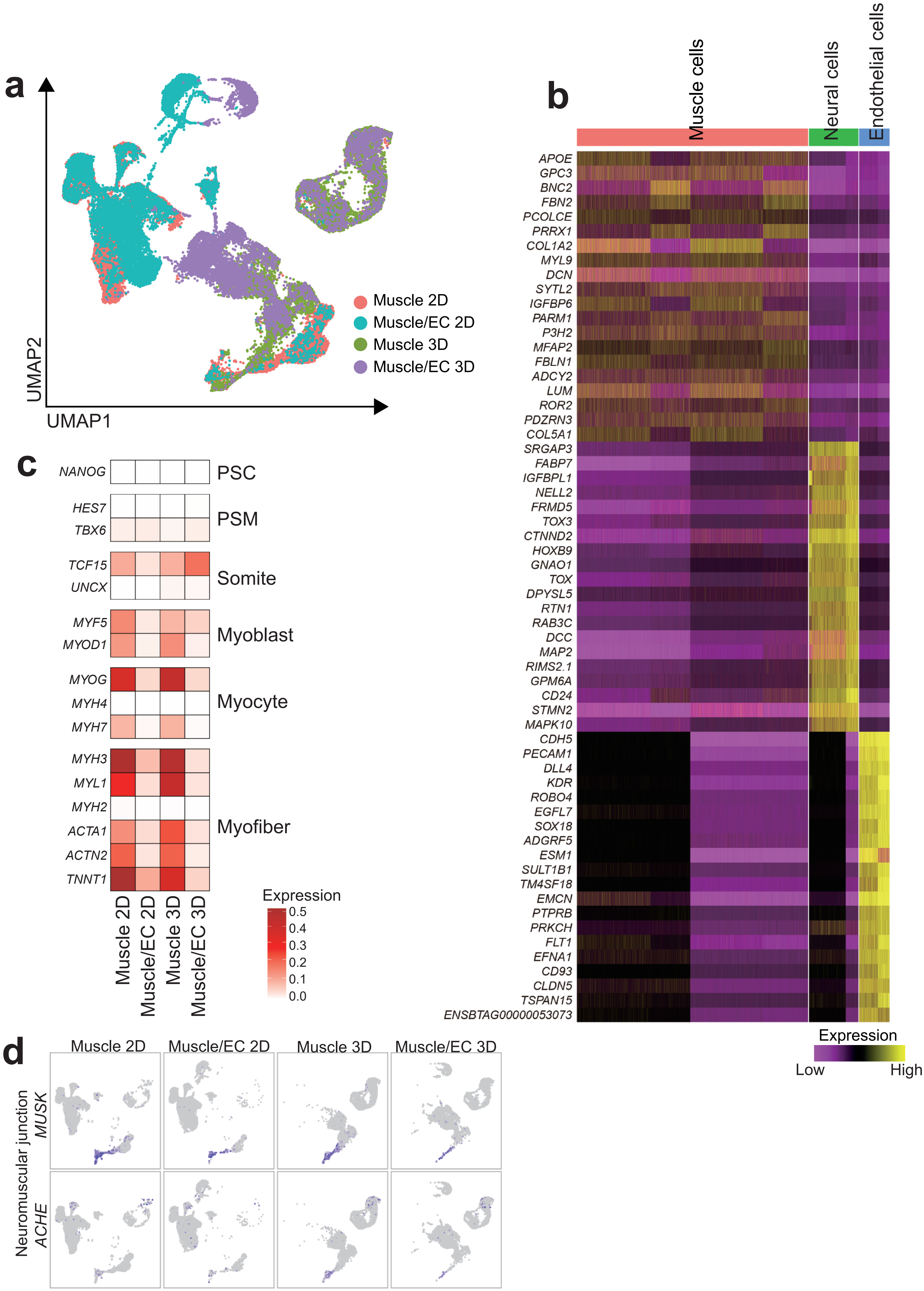

a
UMAP2
UMAP1
Muscle 2D
Muscle/EC 2D
Muscle 3D
Muscle/EC 3D
b
Muscle cells
Neural cells
Endothelial cells
APOE
GPC3
BNC2
FBN2
PCOLCE
PRRX1
COL1A2
MYL9
DCN
SYTL2
IGFBP6
PARM1
P3H2
MFAP2
FBLN1
ADCY2
LUM
ROR2
PDZRN3
COL5A1
SRGAP3
FABP7
IGFBPL1
NELL2
FRMD5
TOX3
CTNND2
HOXB9
GNAO1
TOX
DPYSL5
RTN1
RAB3C
DCC
MAP2
RIMS2.1
GPM6A
CD24
STMN2
MAPK10
CDH5
PECAM1
DLL4
KDR
ROBO4
EGFL7
SOX18
ADGRF5
ESM1
SULT1B1
TM4SF18
EMCN
PTPRB
PRKCH
FLT1
EFNA1
CD93
CLDN5
TSPAN15
ENSBTAG00000053073
Expression
Low
High
c
NANOG
PSC
HES7
TBX6
PSM
TCF15
UNCX
Somite
MYF5
MYOD1
Myoblast
MYOG
MYH4
MYH7
Myocyte
MYH3
MYL1
MYH2
ACTA1
ACTN2
TNNT1
Myofiber
Muscle 2D
Muscle/EC 2D
Muscle 3D
Muscle/EC 3D
Expression
0.5
0.4
0.3
0.2
0.1
0.0
d
Muscle 2D
Muscle/EC 2D
Muscle 3D
Muscle/EC 3D
Neuromuscular junction
MUSK
ACHE

Supplementary Figure 7 scRNA-seq analyses of 4 induction protocols

a, UMAP plot of the integrated data colored by the 4 samples corresponding to individual induction protocols. **b,** Heatmap of 3 identified clusters with the scaled expression of top marker genes. **c,** Heatmap of the expression of selected marker genes for muscle differentiation steps. Color range indicates pseudo-log10 expression levels of SCTransform values. **d,** UMAP plots colored by the expression of selected NMJ marker genes.

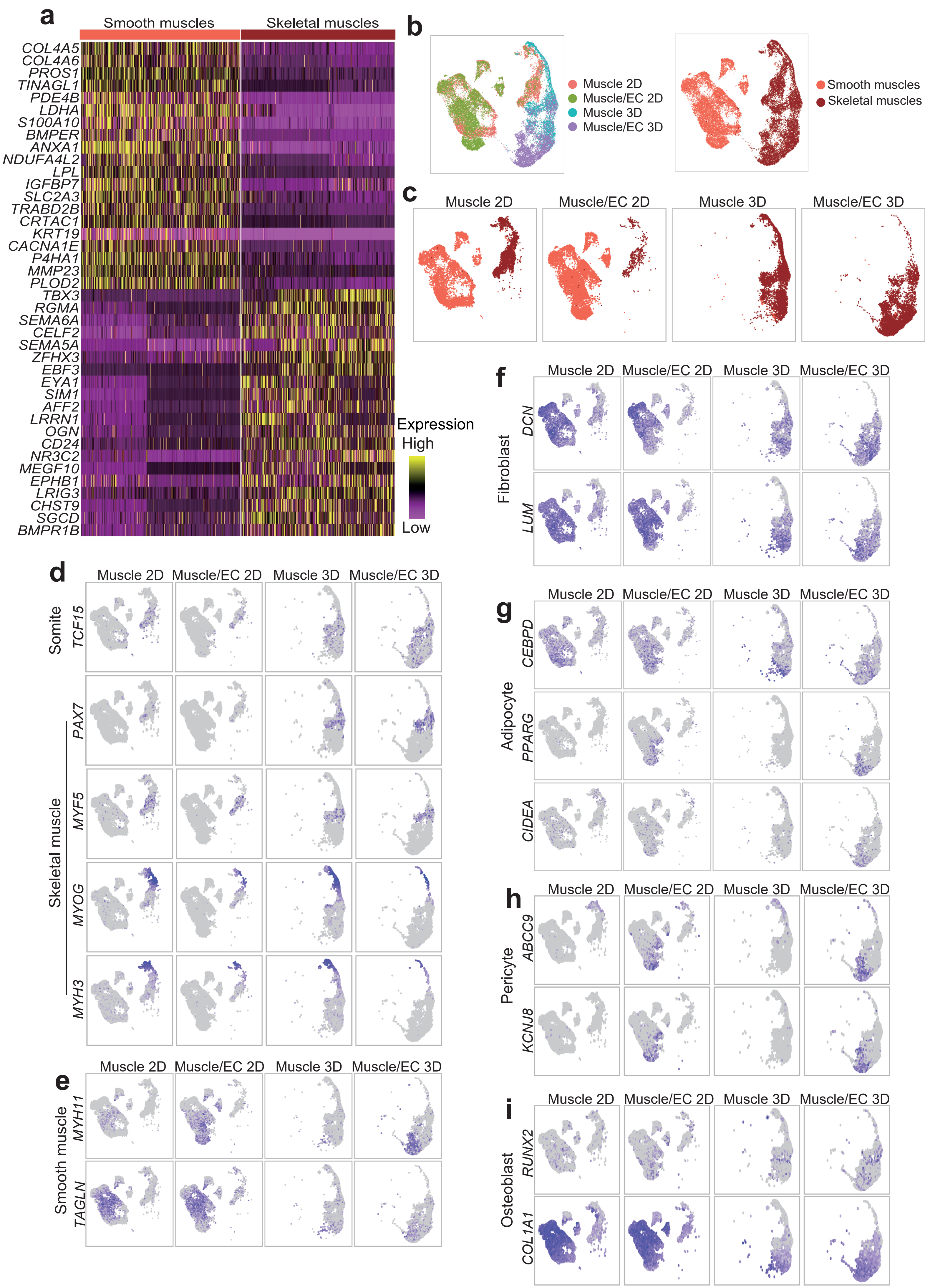

a
Smooth muscles
Skeletal muscles
COL4A5
COL4A6
PROS1
TINAGL1
PDE4B
LDHA
S100A10
BMPER
ANXA1
NDUFA4L2
LPL
IGFBP7
SLC2A3
TRABD2B
CRTAC1
KRT19
CACNA1E
P4HA1
MMP23
PLOD2
TBX3
RGMA
SEMA6A
CELF2
SEMA5A
ZFHX3
EBF3
EYA1
SIM1
AFF2
LRRN1
OGN
CD24
NR3C2
MEGF10
EPHB1
LRIG3
CHST9
SGCD
BMPR1B
Expression
High
Low
b
Muscle 2D
Muscle/EC 2D
Muscle 3D
Muscle/EC 3D
Smooth muscles
Skeletal muscles
c
Muscle 2D
Muscle/EC 2D
Muscle 3D
Muscle/EC 3D
d
Somite
TCF15
Skeletal muscle
PAX7
MYF5
MYOG
MYH3
e
Smooth muscle
MYH11
TAGLN
f
Fibroblast
DCN
LUM
g
Adipocyte
CEBPD
PPARG
CIDEA
h
Pericyte
ABCC9
KCNJ8
i
Osteoblast
RUNX2
COL1A1

Supplementary Figure 8 Subset analyses of the muscle cluster

a, Heatmap of 2 identified subclusters of the muscle cluster with the scaled expression of top marker genes.
b, UMAP plot of the integrated data colored by 4 samples (left) and 2 muscle subclusters (right).
c, Split UMAP plots of the 4 samples corresponding to individual induction protocols. **d-i,** UMAP plots colored by the expression of selected marker genes for skeletal muscle (d), smooth muscle (e), fibroblast (f), adipocyte (g), pericyte (h), and osteoblast (i).

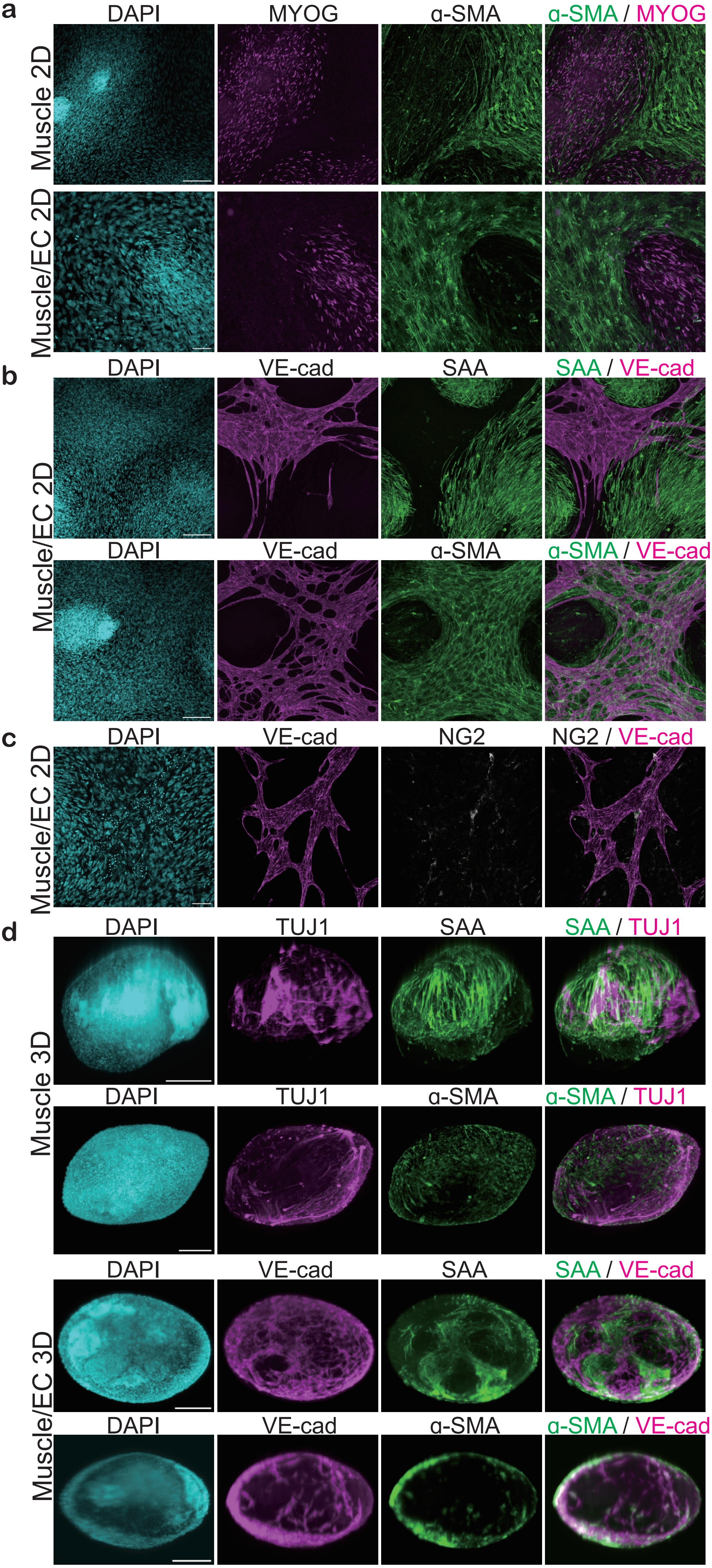

a
DAPI
MYOG
α-SMA
α-SMA / MYOG
Muscle 2D
Muscle/EC 2D
b
DAPI
VE-cad
SAA
SAA / VE-cad
DAPI
VE-cad
α-SMA
α-SMA / VE-cad
Muscle/EC 2D
c
DAPI
VE-cad
NG2
NG2 / VE-cad
Muscle/EC 2D
d
DAPI
TUJ1
SAA
SAA / TUJ1
DAPI
TUJ1
α-SMA
α-SMA / TUJ1
Muscle 3D
DAPI
VE-cad
SAA
SAA / VE-cad
DAPI
VE-cad
α-SMA
α-SMA / VE-cad
Muscle/EC 3D

Supplementary Figure 9 IHC characterization of cell types induced by individual protocols

a, Max projection images of the day-15 culture of the muscle or muscle/EC 2D protocol. MYOG and α-SMA are myocyte and smooth muscle markers, respectively. 20 (Muscle 2D) and 2 (Muscle/EC 2D) independent experiments showed similar staining patterns. **b,** Max projection images of the day-15 culture of the muscle/EC 2D protocol. VE-cad, SAA, and α-SMA are endothelial cell, muscle fiber, and smooth muscle markers, respectively. 10-15 independent experiments showed similar staining patterns. **c,** Max projection images of the day-15 culture of the muscle/EC 2D protocol. VE-cad and NG2 are endothelial cell and pericyte markers, respectively. 9 independent experiments showed similar staining patterns. **d,** Max projection images of the day-15 aggregate of the muscle or muscle/EC 3D protocol. VE-cad, SAA, α-SMA, and TUJ1 are endothelial cell, muscle fiber, smooth muscle markers, and neuronal markers, respectively. 16 (Muscle 3D SAA/TUJ1), 7 (Muscle 3D α-SMA/TUJ1), 15 (Muscle/EC 3D SAA/VE-cad), and 5 (Muscle/EC 3D α-SMA/VE-cad) independent experiments showed similar staining patterns. The max projection of muscle 3D protocol used all Z-stacks, and that of muscle/EC 3D protocol used the Z-stacks within the 40 μm range around the central slice. Scale bars: 50 μm (a, c) and 150 μm (b, d).

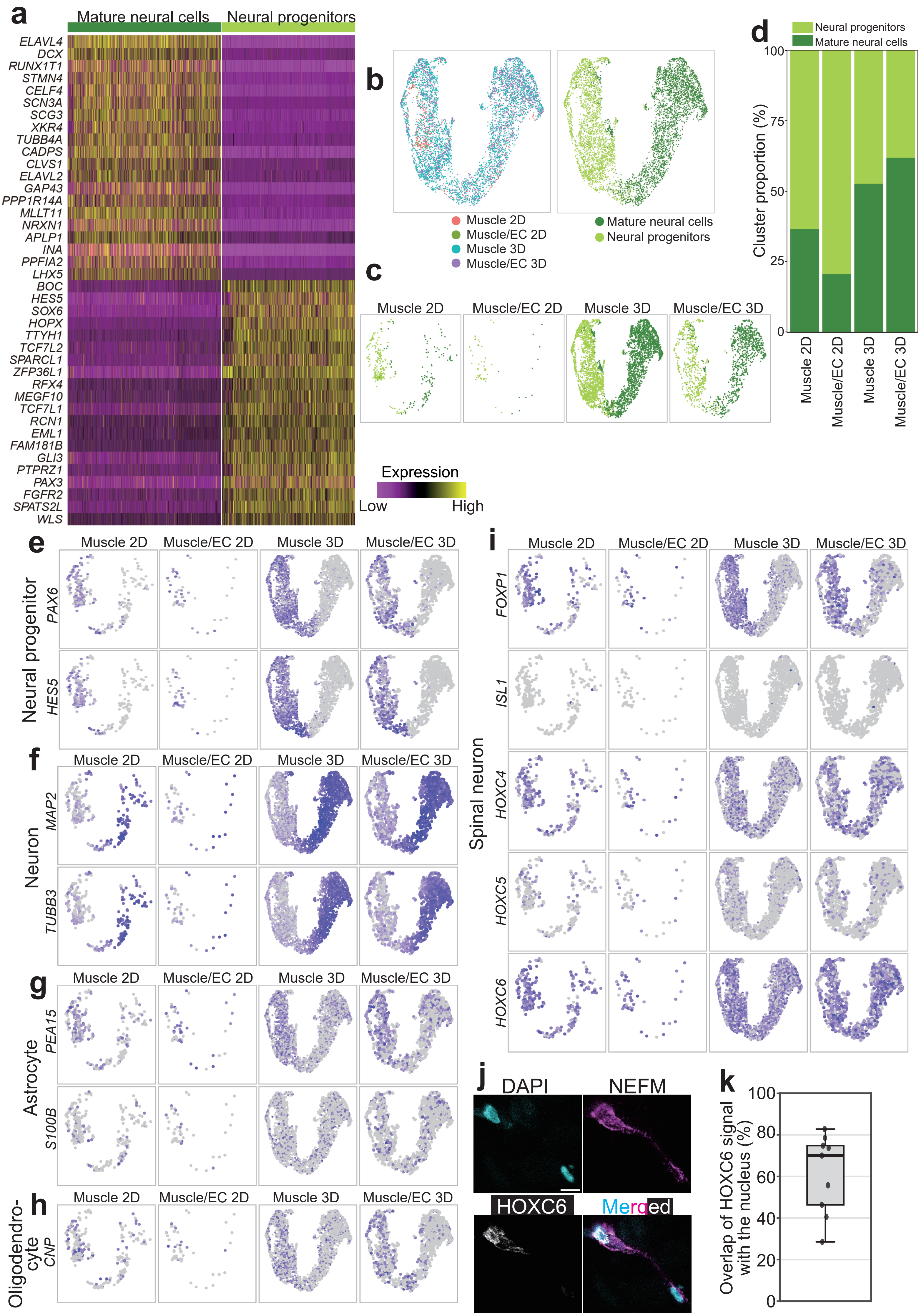

a
Mature neural cells
Neural progenitors
ELAVL4
DCX
RUNX1T1
STMN4
CELF4
SCN3A
SCG3
XKR4
TUBB4A
CADPS
CLVS1
ELAVL2
GAP43
PPP1R14A
MLLT11
NRXN1
APLP1
INA
PPFIA2
LHX5
BOC
HES5
SOX6
HOPX
TTYH1
TCF7L2
SPARCL1
ZFP36L1
RFX4
MEGF10
TCF7L1
RCN1
EML1
FAM181B
GLI3
PTPRZ1
PAX3
FGFR2
SPATS2L
WLS
Expression
Low
High
b
Muscle 2D
Muscle/EC 2D
Muscle 3D
Muscle/EC 3D
Mature neural cells
Neural progenitors
c
Muscle 2D
Muscle/EC 2D
Muscle 3D
Muscle/EC 3D
d
Neural progenitors
Mature neural cells
Cluster proportion (%)
100
75
50
25
0
Muscle 2D
Muscle/EC 2D
Muscle 3D
Muscle/EC 3D
e
Neural progenitor
PAX6
HES5
f
Neuron
MAP2
TUBB3
g
Astrocyte
PEA15
S100B
h
Oligodendrocyte
CNP
i
Spinal neuron
FOXP1
ISL1
HOXC4
HOXC5
HOXC6
j
DAPI
NEFM
HOXC6
Merged
k
Overlap of HOXC6 signal with the nucleus (%)
100
80
60
40
20
0

Supplementary Figure 10 Subset analyses of the neural cluster

a, Heatmap of 2 identified subclusters of the neural cluster with the scaled expression of top marker genes. **b,** UMAP plot of the integrated data colored by 4 samples (left) and 2 neural subclusters (right). **c,** Split UMAP plots of the 4 samples corresponding to individual induction protocols. **d,** Proportions of the 2 neural subclusters in individual samples. **e-i,** UMAP plots colored by the expression of selected marker genes for neural progenitor (e), neuron (f), astrocyte (g), oligodendrocyte (h), and spinal neuron (i). **j,** IHC images of the day-15 culture of the muscle 2D protocol. NEFM and HOXC6 are neurofilament and spinal neuron markers, respectively. 10 independent experiments showed similar staining patterns. Scale bar: 10 μm. **k,** Overlap of the HOXC6 signal with the nuclear compartment. Boxplot indicates the median, 25th percentile, and 75th percentile. Whiskers extend to the minimum and maximum non-outlier values. N = 9 cells with the entire cell morphology visible, from 10 independent experiments. Source data are provided as a Source Data file.
