# Supplementary material for: Self-organization of vascularized muscle from bovine embryonic stem cells
Source: Nat Commun. 2026 Sep 2;17:8975. doi: 10.1038/s41467-026-76569-2 (PMC13538390; doi:10.1038/s41467-026-76569-2)
Supplement: Supplementary file 3 [file 41467_2026_76569_MOESM2_ESM.pdf]

nature portfolio

# Peer Review File

# Self-organization of vascularized muscle from bovine embryonic stem cells

Corresponding Author: Professor Miki Ebisuya

**This file contains all reviewer reports in order by version, followed by all author rebuttals in order by version.**

Version 0:

Reviewer comments:

Reviewer #1

(Remarks to the Author)
The authors have the ambitious goal of establishing protocols for generating self-organized, vascularized skeletal muscle from bovine embryonic stem cells (ESCs), aiming at the production of artificial meat. The proposed approach distinguishes itself from previously established approaches due to the utilization of the principle of self-organization, enabling progenitor cells derived from bovine ESCs to co-differentiate into muscle, neuronal, and endothelial cells. While the initial findings are intriguing, the manuscript perhaps overstates the readiness of these protocols for application in meat production. The complexity of creating a steak, which is a large and complex tissue, presents significant challenges that are not fully addressed in the current submission. Key issues such as the scalability of production, cost-efficiency of differentiation processes, consistency in quality and taste, and the expense associated with growth media are touched upon but not sufficiently explored. These are critical factors that could significantly impact the feasibility and practicality of the proposed method in a commercial setting. Moreover, the manuscript does not discuss the generation and integration of fat and connective tissues, which are crucial for replicating the authentic taste and texture of steak. The absence of these components raises questions about the potential applicability of the proposed method.

Furthermore, the manuscript divides its methodology into four distinct sections, each dedicated to the generation of different cell types from bovine embryonic stem cells. This structure is logical, yet the characterization of these tissues and the quantification of their functionality and reproducibility require further elaboration. Specifically, the significance and advantage of incorporating endothelial cells and neurons within the muscle tissue culture are not clearly justified. Intuitively, one might argue that the inclusion of fat cells would be more directly relevant to the qualities of steak production. In conclusion, while the study describes the generation of muscles, endothelial cells, and neurons from bovine embryonic stem cells, a more comprehensive characterization of the models is required.

Major concerns:

Induction of PSM and muscle fibers
- For the presentation of the data, the authors should use different symbol shapes or colors for the different experiments to show the robustness of the proposed differentiation protocol. As the protocol is performed with one cell line, the differentiation of the different experiments in the data presented is essential.

Co-induction of bovine muscle and spinal neurons
- The authors state that muscle and neurons are intermixed in the cultures, but leave open whether there is a direct interaction between the two cell types. This analysis would confirm whether the neurons present in the culture actually form synaptic connections with the muscle fibers, demonstrating the direct interaction between the two cell types.
- In addition, assessment of the density and distribution of AChRs at the NMJ by α-bungarotoxin staining would provide information on the maturity and functionality of the neuromuscular junctions in the artificial muscle tissue.

Co-induction of bovine muscle and endothelial cells
- In the images presented by the authors, the muscle area in the vascularized muscle appears smaller than in the non-vascularized muscle. The authors should discuss how the induction of endothelial cells affects other cell types. Therefore, quantification of muscle area in non-vascularized and vascularized muscle should be considered.
- Presence of neurons in the vascularized culture: As the vascularized protocol is an evolution of the previous one, it is

important to determine whether neurons are present in the vascularized culture. Furthermore, assessing whether the muscle fibers contract in the presence of neurons in these cultures would indicate the functional maturation of the system.
- Effect of vascularization on maturation: It is important to understand how vascularization affects the maturation of the system. To do this, parameters such as culture duration, maturation state and the potential for muscle contraction need to be assessed. Investigating whether the presence of endothelial cells has a positive effect on these aspects would determine the role of vascularisation in the development of artificial muscle tissue.

Induction of bovine muscle fibers in 3D
- Scalability: The authors should discuss the scalability of their model in terms of size, yield and achievable maturity. Factors such as the efficiency of production, the time required for maturation and the potential meat yield of the final product should be considered. Comparisons with previous methods of artificial meat production would provide context for assessing the scalability of the model presented.
- Quantification of muscle size: Quantifying the muscle size of vascularized and non-vascularized organoids is crucial as reduced muscle mass can significantly affect the yield of meat production. This analysis would help to understand the potential limitations of the model and identify optimization opportunities to improve meat yield.
- Serum-free protocol: Although the authors claim their protocol is serum-free, the use of Matrigel derived from mouse carcinoma cells raises concerns about animal components. To adhere to an animal component-free protocol, the authors should discuss alternatives to Matrigel that are available and suitable for use in their model. This discussion would increase the transparency of the protocol and its potential for wider application in the field of cultured meat production.

Reviewer #2

(Remarks to the Author)
I co-reviewed this manuscript with one of the reviewers who provided the listed reports. This is part of the Nature Communications initiative to facilitate training in peer review and to provide appropriate recognition for Early Career Researchers who co-review manuscripts.

Reviewer #3

(Remarks to the Author)
Sanaki-Matsumiya et al. developed two protocols to convert bovine ESCs into 1) skeletal muscle as well as neuronal cells, and 2) skeletal muscle cells along with endothelial cells. They implemented both methods in 2D and 3D cultures, ultimately aiming to produce lab-grown beef replete with innervation and vascularization. It was striking that the bovine muscle cells displayed calcium oscillations and a bovine segmentation clock of about 4h. The 2D muscle+endothelial co-induction protocol also yielded largely SAA+ skeletal muscle and VE-cad+ endothelial populations. This is an innovative approach with obvious practical applications.

While the manuscript is compelling, the authors can consider the following suggestions to further enhance the study:

1. Scalability is a key aspect of lab-grown beef production. How many skeletal muscle cells/endothelial cells/neuronal cells can 1 hPSC yield after 15 days using the 2D and 3D protocols?
2. The 2D muscle + neuronal co-induction protocol generated some cells that are neither SAA+ nor TUJI+. What are those SAA- TUJI- cells? What is the purity of the cells generated? Information on the purity or proportions of each cell type would be valuable for researchers looking to replicate the study.
3. Vascularization of bovine muscle organoids was shown by the presence of VE-cad+ cells. What endothelial subtype was generated? Are these endothelial cells arterial or venous?
4. The identification of spinal neurons was based on two markers (e.g., TUJ1 and SMI-32). So, it is unclear whether these neurons are indeed “spinal,” as the authors claimed. The authors could assess additional markers to confirm the cellular identity of the neurons. For markers of spinal (motor) neurons, please see https://www.nature.com/articles/s41467-022-35574-x
5. In the discussion section, elaborating on how the mixed signals used to make two cell types would provide deeper insights into how the cells were generated.
6. Finally, since skeletal muscle, endothelial, and neuronal cells were generated from two different protocols, it would be beneficial for the authors to address how future work needs to integrate these cell types into a single culture system.

Version 1:

Reviewer comments:

Reviewer #1

(Remarks to the Author)
The authors have performed additional experiments to characterize their in vitro models further. However, several critical concerns remain unresolved and must be addressed before the manuscript can be considered for publication in Nature Communications. Unfortunately, the revised version does not provide sufficient evidence to support the main conclusions,

raising significant concerns.
- In the abstract, the authors claim that neurons and muscles self-organize in their model. However, based on the provided immunofluorescence images, there is no clear evidence that the 3D structures self-organize. The neurons and muscles appear intermixed without signs of spatial organization. The authors should revise their claim to reflect their findings more accurately or provide compelling evidence of distinct compartmentalization.

- Insufficient Evidence for Neuromuscular Junction (NMJ) Formation (Lines 114–116). The authors report that blocking calcium transients in muscle cells is sufficient evidence to prove NMJ formation. We strongly disagree with this conclusion. α-Bungarotoxin (α-BTX) staining is a well-established and widely accepted marker of postsynaptic acetylcholine receptor (AChR) clustering, which is critical for confirming NMJ formation. The absence of α-BTX staining suggests that functional NMJs have not fully developed in this model, as AChRs are likely not clustering at the postsynaptic membrane. If presynaptic terminals fail to make stable contact with muscle fibers, postsynaptic differentiation (AChR clustering) will not occur. Curare alone is not sufficient to prove NMJ formation unless there is additional evidence of AChR clustering. To address this, the authors must: Provide α-BTX staining in the revised version. If NMJs are not convincingly demonstrated, the authors should revise their claims to indicate the presence of neurons and muscle cells rather than functional NMJs. In the discussion, the authors may state that future work will focus on optimizing conditions for NMJ formation.

- Additionally, the manuscript does not justify why cultures are only maintained for 15 days. It is possible that this timeline is too short for NMJs to mature. The authors should discuss whether longer culture durations might improve NMJ formation. Are the authors observing the formation of a necrotic core and for that reason they can not maintain the 3D cultures longer? If yes, this information should be included in the manuscript.

- The requested analysis of individual experiments reveals batch effects that influence the curare results. It seems that only one experiment showed some contraction (exp2) but not the other two experiments (exp1 and exp3). The authors should provide separate data for each batch with and without curare treatment to improve clarity and reproducibility. Please clearly discuss any batch-to-batch variability observed in your experiments. Unfortunately, these data are not convincing to support the formation of NMJs in the model.

- The authors must clarify whether their neurons are spinal cord motor neurons before making conclusions about their differentiation pathway (Lines 120-122). The authors do not provide evidence that their neurons possess a spinal cord motor neuron identity.They must use specific Hox markers and immunofluorescence analysis to support their claims to determine whether their neurons have a spinal cord or hindbrain identity. If the neurons originate from neuromesodermal progenitors, they should acquire a spinal cord identity. If the immunofluorescence analysis reveals hindbrain identity, the authors must revise their claims in Lines 120–122 and remove the assertion that their neurons originate from NMPs.

- The quantification in Figure 6c summarizes the presence of neural, endothelial, and muscle cells from single-cell RNA sequencing data. However, it is concerning that smooth muscle cells appear highly prominent in the 2D and 2D/EC models, while the skeletal muscle cluster is a minor population. Since the goal is cultured beef production, the focus should be on skeletal muscle development rather than smooth muscle. To improve clarity, the authors must plot the percentage of smooth muscle vs. skeletal muscle in each model. Please clearly distinguish between skeletal and smooth muscle cells for each condition in the revised version.

- Thank you for providing a video of beating 3D cultures. However, the contractions are only occasionally observed and are not clearly defined. To strengthen their findings, the authors could: (i) Induce contractions via acetylcholine stimulation to determine if neuromuscular activity can be enhanced. (ii) Improve video documentation to clearly illustrate contraction events.

- To improve the manuscript's impact, the authors should clearly state which of their models is the most suitable for cultured meat production based on:
a) Muscle content/yield
b) Cost-effectiveness
c) Reproducibility

This would help guide future applications of their work in meat production.

- The authors should acknowledge in their discussion that their bovine 3D meat cultures currently rely on ECM derived from mouse carcinoma cells. While this may be necessary for now, future efforts should focus on developing an alternative, animal-free ECM for commercial viability and regulatory approval.

By addressing these concerns, the manuscript will be significantly stronger, more rigorous, and better aligned with its claims.

Reviewer #2

(Remarks to the Author)
I co-reviewed this manuscript with one of the reviewers who provided the listed reports. This is part of the Nature Communications initiative to facilitate training in peer review and to provide appropriate recognition for Early Career Researchers who co-review manuscripts.

Reviewer #3

(Remarks to the Author)
The authors have addressed most of my comments in their revision. Adding single-cell RNA sequencing data to quantify cell type proportions significantly strengthens the manuscript and provides additional definitive insights into differentiation outcomes. I have no further major comments.

Remaining minor concerns:

The expression of the pericyte marker of KCNJ8 and ABCC9 overlaps with the expression of smooth muscle-specific markers (MYH11), suggesting that the population could be smooth muscle cells (Supplemental Figure 7i).

The manuscript would benefit from a more thorough discussion regarding the reproducibility of the described protocols across different bovine embryonic stem cell lines.

The scRNA-seq analysis reveals a predominance of smooth muscle cell populations, with a relatively smaller fraction expressing skeletal muscle cell markers. This suggests a deviation from the original objective of generating skeletal muscle cells. I recommend the authors conduct further analysis of their existing data and/or discuss this observation that future protocols can further enhance skeletal muscle differentiation efficiency. This would better align the results with the overall goals of the study.

Version 2:

Reviewer comments:

Reviewer #1

(Remarks to the Author)
In this manuscript, Sanaki-Matsumiya et al. present a protocol for the co-induction of skeletal muscle, neuronal, and endothelial lineages from bovine embryonic stem cells, resulting in the formation of multicellular 2D and 3D tissue-like structures. The study is technically interesting and relevant to both organoid biology and cultured meat applications. The use of serum-free conditions, the relatively rapid differentiation, and the inclusion of single-cell RNA sequencing add significant value to the work.
The revised manuscript has improved in several aspects, including additional staining, functional assays, and a more transparent analysis of cell-type composition. However, concerns remain regarding the interpretation of neuromuscular junction formation, functional maturity, and reproducibility. Addressing these points would further strengthen the manuscript and better align the conclusions with the data.

Major comments
The evidence for neuromuscular junction (NMJ) formation remains limited. While the addition of α-bungarotoxin staining and expanded calcium imaging experiments is appreciated, the data do not yet convincingly demonstrate fully functional NMJs. The α-BTX signal appears as punctate staining without quantitative analysis of acetylcholine receptor clustering or clear co-localization with presynaptic markers. In addition, curare-sensitive calcium oscillations, while suggestive, are not sufficient on their own to establish functional neuromuscular synapses, and muscle contractions remain inconsistent and were not included. Given that α-BTX staining can also produce non-specific punctate background, the interpretation is further complicated. It would therefore be appropriate to temper the claims and refer to these structures as early NMJ-like interactions. Quantification of α-BTX-positive clusters, ideally correlated with functional readouts such as curare response in the same samples, would substantially strengthen the conclusions.

The additional experiments addressing curare sensitivity are helpful, but they reveal notable inter-batch variability. The reported consistent differences between treated and untreated samples are not uniformly evident across all experiments, and this variability is largely obscured in the summarized presentation. Presenting the batch-wise data more prominently would improve transparency. In addition, the statistical approach should be more clearly described and justified, particularly with regard to the treatment of biological replicates versus regions of interest and the handling of inter-batch variability. A brief discussion of possible sources of variability, such as differences in differentiation efficiency, neuronal density, or maturation state, would also be important.

The extension of cultures to day 30 without evidence of necrosis is informative; however, the lack of observable maturation between day 15 and day 30 raises important questions about the developmental potential of the system. Early NMJs do not appear to mature over time, and muscle contractions remain weak and inconsistent. The manuscript would benefit from a more explicit discussion of the factors that may limit maturation in this system, such as the absence of activity-dependent cues, appropriate chemical signals, Schwann cells, etc., and how these limitations affect the interpretation of the model.

The characterization of neuronal identity has been strengthened by the inclusion of HOX gene expression and motor neuron markers. However, the HOXC6 immunostaining does not appear exclusively nuclear, which raises some uncertainty regarding its interpretation. Clarifying this point or further tempering the statement would improve the manuscript.

The single-cell RNA sequencing analysis and additional staining reveal a substantial contribution of smooth muscle cells, particularly in 2D conditions, with skeletal muscle being less dominant. While the authors appropriately highlight that 3D

cultures are more favorable, this observation points to limitations in lineage specificity and control within the differentiation protocol. A more explicit discussion of the biological basis for the differences between 2D and 3D conditions would help explain the observed differences, as well as potential strategies to improve skeletal muscle specification. This also raises concerns regarding the current suitability of the system for skeletal muscle-focused applications.

Given the broader muscle composition observed, the authors may wish to consider whether the current title fully reflects the scope of the study, and whether replacing “skeletal muscle” with “muscle” would provide a more accurate description.

Finally, the use of a single bovine ESC line limits generalizability, and although this is acknowledged, it remains an important consideration related to the reproducibility and scalability of the approach.

Version 3:

Reviewer comments:

Reviewer #1

(Remarks to the Author)

The authors have satisfactorily addressed my previous concerns, and the revised manuscript has been significantly strengthened. I am happy to support the publication of this manuscript in Nature Communications.

**Open Access** This Peer Review File is licensed under a Creative Commons Attribution 4.0 International License, which permits use, sharing, adaptation, distribution and reproduction in any medium or format, as long as you give appropriate credit to the original author(s) and the source, provide a link to the Creative Commons license, and indicate if changes were made.
In cases where reviewers are anonymous, credit should be given to 'Anonymous Referee' and the source.
The images or other third party material in this Peer Review File are included in the article’s Creative Commons license, unless indicated otherwise in a credit line to the material. If material is not included in the article’s Creative Commons license and your intended use is not permitted by statutory regulation or exceeds the permitted use, you will need to obtain permission directly from the copyright holder.
To view a copy of this license, visit https://creativecommons.org/licenses/by/4.0/

**In reply to Reviewer 1:**

The authors have the ambitious goal of establishing protocols for generating self-organized, vascularized skeletal muscle from bovine embryonic stem cells (ESCs), aiming at the production of artificial meat. The proposed approach distinguishes itself from previously established approaches due to the utilization of the principle of self-organization, enabling progenitor cells derived from bovine ESCs to co-differentiate into muscle, neuronal, and endothelial cells.

While the initial findings are intriguing, the manuscript perhaps overstates the readiness of these protocols for application in meat production. The complexity of creating a steak, which is a large and complex tissue, presents significant challenges that are not fully addressed in the current submission. Key issues such as the scalability of production, cost-efficiency of differentiation processes, consistency in quality and taste, and the expense associated with growth media are touched upon but not sufficiently explored. These are critical factors that could significantly impact the feasibility and practicality of the proposed method in a commercial setting. Moreover, the manuscript does not discuss the generation and integration of fat and connective tissues, which are crucial for replicating the authentic taste and texture of steak. The absence of these components raises questions about the potential applicability of the proposed method.

1-1. Although our manuscript focuses on proof of principle rather than the application stage, we fully agree with the reviewer's suggestion to discuss the challenges and limitations associated with implementing our protocols in meat production.

- Scalability and cost

Bovine ESCs can proliferate rapidly and indefinitely – they are split every other day at 1/5-1/10 dilution. Under the 2D differentiation protocols, the cell number increases by 50- to 80-fold in 15 days. Under the 3D protocols, an aggregate derived from 20,000 ES cells develops into a 0.1 $mm^3$ (~0.11 mg) muscle spheroid in 15 days.

In contrast, the current cost is clearly too high for commercialization: one gram of muscle aggregates costs 3,611 (muscle 3D protocol) or 8,676 (muscle/EC 3D protocol) EUR. Since the primary expenses arise from growth factors, chemical inhibitors, and Matrigel, mass-producing these products or finding cost-effective alternatives is crucial.

We discussed these points in the discussion section and new Supplementary Table 1.

- Taste and texture

To investigate the potential for generating fat and connective tissues, we performed single-cell RNA sequencing (scRNA-seq) analyses (new Supplementary Fig. 7). Fibroblast and extracellular matrix markers, DCN and LUM, were detected in the muscle cell cluster, suggesting the presence of fibroblasts connecting muscle cells. While mature fat cells were not explicitly detected, several adipocyte progenitor markers, CEBPD, PPARG, and CIDEA, were also detected, suggesting a potential modification of the protocol to co-induce adipocytes.

Furthermore, the manuscript divides its methodology into four distinct sections, each dedicated to the generation of different cell types from bovine embryonic stem cells. This structure is logical, yet the characterization of these tissues and the quantification of their functionality and reproducibility require further elaboration. Specifically, the significance and advantage of incorporating endothelial cells and neurons within the muscle tissue culture are not clearly justified. Intuitively, one might argue that the inclusion of fat cells would be more directly relevant to the qualities of steak production. In conclusion, while the study describes the generation of muscles, endothelial cells, and neurons from bovine embryonic stem cells, a more comprehensive characterization of the models is required.

1-2. We acknowledge the need for a more comprehensive characterization of our models. To address this, we performed scRNA-seq analyses on the four distinct models (new Fig. 6; new Supplementary Fig. 6-8), with the details discussed in the relevant sections below.

Our decision to co-induce endothelial cells rather than fat cells was driven by our aim to explore the self-organization potential of ESC-derived organoids. The lack of vasculature is a significant challenge in developing steak-like tissues, and enabling the self-organization of blood vessels is a critical frontier in organoid research. Neurons may be beneficial for muscle maturation, potentially offering an alternative to the external electrical. As noted earlier, co-inducing fat cells also looks promising and will be an important focus for future studies.

Major concerns:
Induction of PSM and muscle fibers
- For the presentation of the data, the authors should use different symbol shapes or colors for the different experiments to show the robustness of the proposed differentiation protocol. As the protocol is performed with one cell line, the differentiation of the different experiments in the data presented is essential.
1-3. Following the suggestion, we changed the colors for individual experiments in all relevant figures (new Fig. 1g,h; 2c; 3c,f; 4c,f,g; 5c,i,j).

Co-induction of bovine muscle and spinal neurons
- The authors state that muscle and neurons are intermixed in the cultures, but leave open whether there is a direct interaction between the two cell types. This analysis would confirm whether the neurons present in the culture actually form synaptic connections with the muscle fibers, demonstrating the direct interaction between the two cell types.
- In addition, assessment of the density and distribution of AChRs at the NMJ by α-bungarotoxin staining would provide information on the maturity and functionality of the neuromuscular junctions in the artificial muscle tissue.
1-4. We think that the inhibition of calcium pulses by an acetylcholine receptor blocker, curare demonstrated the functionality of NMJs (new Fig. 2b,c).
Furthermore, our scRNA-seq data showed the expression of NMJ markers MUSK and ACHE in muscle and neural clusters (new Supplementary Fig. 6d).

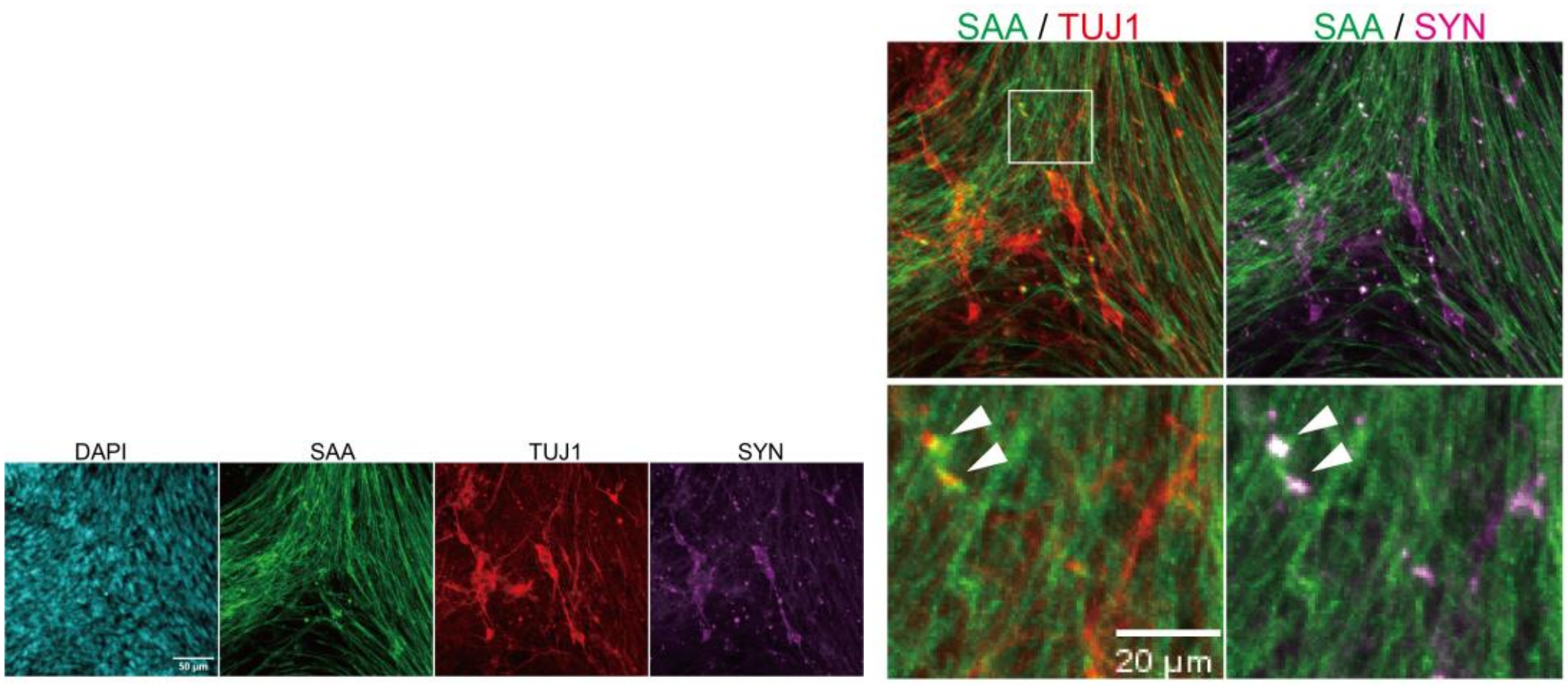


Additional Data 1
Synaptophysin (SYN)-positive synapses were detected around TUJ1-positive neurons and SAA-positive muscles in the day-15 culture under the muscle 2D protocol. White arrowheads indicate potential NMJs.

We also stained synapses (see Additional Data 1). The synaptophysin antibody detects both NMJs and neuron-neuron synapses, and we believe that the triple-positive dots for SAA, TUJ1, and SYN represent NMJs (white arrowheads in Additional Data 1). However, we could not reach a definitive conclusion, so we have included the images only in this letter.

Co-induction of bovine muscle and endothelial cells

- In the images presented by the authors, the muscle area in the vascularized muscle appears smaller than in the non-vascularized muscle. The authors should discuss how the induction of endothelial cells affects other cell types. Therefore, quantification of muscle area in non-vascularized and vascularized muscle should be considered.

1-5. To evaluate the impact of endothelial cell (EC) induction on other cell types, we quantified the proportions of different cell types between the muscle and muscle/EC protocols (new Fig. 6b,c). The presence of ECs led to a decrease in the proportion of neural cells, while the effect on muscle cells was relatively minor. In fact, the difference between 2D and 3D protocols was more pronounced: 3D protocols produced a higher proportion of neural cells.

- Presence of neurons in the vascularized culture: As the vascularized protocol is an evolution of the previous one, it is important to determine whether neurons are present in the vascularized culture. Furthermore, assessing whether the muscle fibers contract in the presence of neurons in these cultures would indicate the functional maturation of the system.

1-6. We apologize for the misleading text. Even under the muscle/EC co-induction protocols, TUJ1-positive neurons were present (new Supplementary Fig. 3; 5a). Our scRNA-seq analyses also detected neural clusters in both 2D and 3D cultures under the muscle/EC co-induction protocols (new Fig. 6b,c; new Supplementary Fig. 8). Note, however, that the proportion of neural cells was lowest under the muscle/EC 2D protocol. We modified the manuscript to explicitly mention the existence of neurons under the muscle/EC protocols.

Muscle contraction was occasionally observed, particularly in 3D cultures (see Additional Video 1). However, these events were inconsistent, making it challenging to reliably compare contraction patterns between the muscle and muscle/EC protocols.

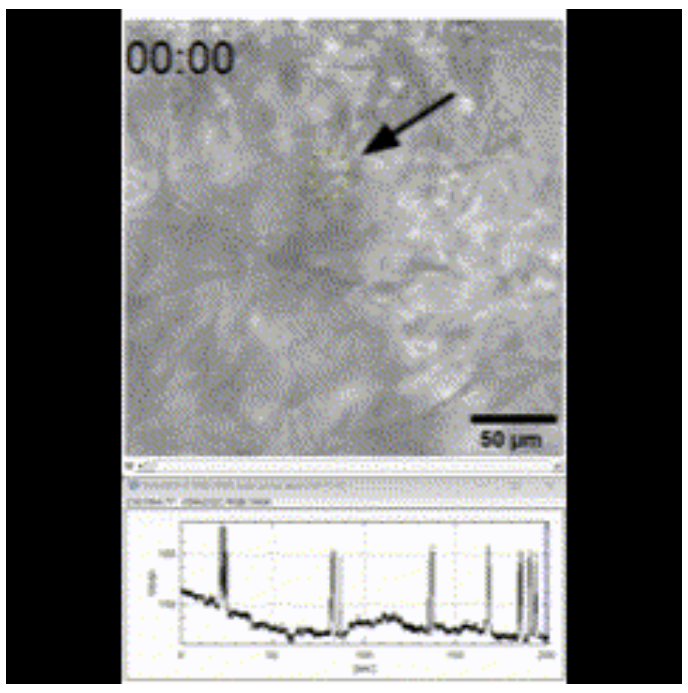


Snapshot of Additional Video 1.mp4
The movie shows spontaneous beatings of muscle fibers.

- Effect of vascularization on maturation: It is important to understand how vascularization affects the maturation of the system. To do this, parameters such as culture duration, maturation state and the potential for muscle contraction need to be assessed. Investigating whether the presence of endothelial cells has a positive effect on these aspects would determine the role of vascularisation in the development of artificial muscle tissue.

1-7. While we fully understand the reviewer's point, we would like to emphasize that co-induction of EC networks is a step towards perfusable vessels and that we do not expect obvious positive effects of having ECs on muscle tissues at this point.

Our scRNA-seq data showed similar muscle maturation steps in all four protocols; in fact, the muscle protocols without EC tended to induce higher expression of mature myofiber markers (new Supplementary Fig. 6c).

Interestingly, several markers of adipocytes, pericytes, and osteoblasts exhibited higher expression levels under the muscle/EC protocols compared to the muscle protocols (new Supplementary Fig. 7h-j; see PPARG, ABCC9, KCNJ8, and RUNX2). This difference can be caused by ECs themselves or by additional stimuli, such as VEGF and Forskolin, in the EC induction protocols.

Induction of bovine muscle fibers in 3D
- Scalability: The authors should discuss the scalability of their model in terms of size, yield and achievable maturity. Factors such as the efficiency of production, the time required for maturation and the potential meat yield of the final product should be considered. Comparisons with previous methods of artificial meat production would provide context for assessing the scalability of the model presented.
1-8. As discussed in point 1-1, an aggregate of 20,000 bovine ES cells develops into a 0.1 $mm^3$ muscle spheroid within 15 days. The clear problem is the cost, which remains as high as that of the first lab-grown hamburger created from myosatellite cells by Dr. Mark Post in 2013. As the cost of myosatellite-derived cultured beef has decreased by over 20,000-fold since then, dramatically reducing the cost of ESC-derived cultured beef should also be achievable.

- Quantification of muscle size: Quantifying the muscle size of vascularized and non-vascularized organoids is crucial as reduced muscle mass can significantly affect the yield of meat production. This analysis would help to understand the potential limitations of the model and identify optimization opportunities to improve meat yield.
1-9. Co-induction of ECs slightly increased the proportion of muscle cells in 3D cultures (new Fig. 6c). As discussed in point 1-5 and 1-7, however, we consider the functionalization of vasculature and positive effects on muscle tissues as the future step. We explicitly mentioned the limitation in the discussion section: “we did not observe a clear positive impact of bovine endothelial networks on muscle cultures at this stage, and further functionalization of the bovine blood vessels and neuromuscular junctions will be necessary”.

- Serum-free protocol: Although the authors claim their protocol is serum-free, the use of Matrigel derived from mouse carcinoma cells raises concerns about animal components. To adhere to an animal component-free protocol, the authors should discuss alternatives to Matrigel that are available and suitable for use in their model. This discussion would increase the transparency of the protocol and its potential for wider application in the field of cultured meat production.

Matrigel coat Lamin coat

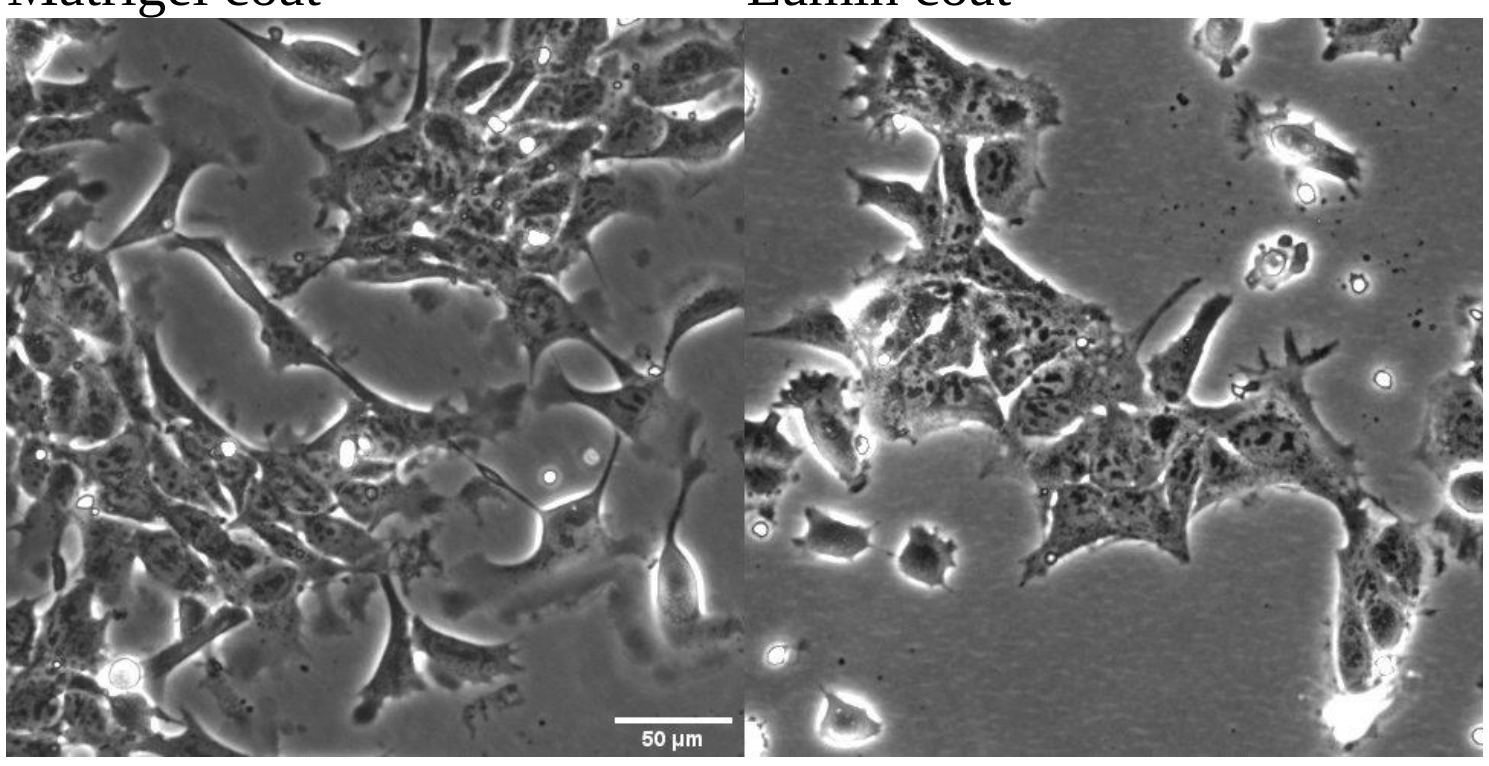


Additional Data 2
Bovine ESCs cultured on Matrigel- or Laminin-coated dishes. On laminin dishes, the cells exhibited morphological changes, including enlarged nuclei, suggesting a loss of pluripotency.

1-10. We agree with the reviewer that Matrigel is not desirable in terms of animal components, product variability, and high cost. Unfortunately, our efforts to identify an effective replacement have been unsuccessful.
Under the 2D differentiation protocols, we tested alternative coating materials such as laminin and gelatin. However, bovine cells were unable to adhere to the dishes until day 15 unless Matrigel-coated dishes were used.
Under the 3D protocols, the aggregates could not maintain the size and structure without low-concentration Matrigel in the medium (new Supplementary Fig. 4).
Even during the maintenance culture of bovine ESCs, Matrigel-coating was essential – ESCs cultured on laminin-coated dishes could not maintain the pluripotency (see Additional Data 2).

**In reply to Reviewer 2:**
I co-reviewed this manuscript with one of the reviewers who provided the listed reports. This is part of the Nature Communications initiative to facilitate training in peer review and to provide appropriate recognition for Early Career Researchers who co-review manuscripts.

**In reply to Reviewer 3:**
Sanaki-Matsumiya et al. developed two protocols to convert bovine ESCs into 1) skeletal muscle as well as neuronal cells, and 2) skeletal muscle cells along with endothelial cells. They implemented both methods in 2D and 3D cultures, ultimately aiming to produce lab-grown beef replete with innervation and vascularization. It was striking that the bovine muscle cells displayed calcium oscillations and a bovine segmentation clock of about 4h. The 2D muscle+endothelial co-induction protocol also yielded largely SAA+ muscle+skeletal muscle and VE-cad+ endothelial populations. This is an innovative approach with obvious practical applications.
While the manuscript is compelling, the authors can consider the following suggestions to further enhance the study:

1. Scalability is a key aspect of lab-grown beef production. How many skeletal muscle cells/endothelial cells/neuronal cells can 1 hPSC yield after 15 days using the 2D and 3D protocols?
3-1. We fully agree with the reviewer's suggestion to discuss the scalability. Bovine ESCs can proliferate rapidly and indefinitely – they are split every other day at 1/5-1/10 dilution. Under the 2D differentiation protocols, the cell number increases by 50- to 80-fold in 15 days. Under the 3D protocols, an aggregate derived from 20,000 ES cells develops into a 0.1 $mm^3$ (~0.11 mg) muscle spheroid in 15 days.
To evaluate the proportions of individual cell types, we performed single-cell RNA sequencing (scRNA-seq) analyses (new Fig. 6; new Supplementary Fig. 6-8). Under the muscle/EC 2D protocol, the proportions of muscle, endothelial, neural cells were 79%, 20%, and 0.6%, respectively. Under the muscle/EC 3D protocol, the proportions of muscle, endothelial, neural cells were 6%, 17%, and 18%, respectively (new Fig. 6c).

In contrast, the current cost is clearly too high for commercialization: one gram of muscle aggregates costs 3,611 (muscle 3D protocol) or 8,676 (muscle/EC 3D protocol) EUR. Since the primary expenses arise from growth factors, chemical inhibitors, and Matrigel, mass-producing these products or finding cost-effective alternatives is crucial.
We discussed these points in the discussion section and new Supplementary Table 1.

2. The 2D muscle + neuronal co-induction protocol generated some cells that are neither SAA+ nor TUJI+. What are those SAA- TUJI- cells? What is the purity of the TUJI+cells generated?

Information on the purity or proportions of each cell type would be valuable for researchers looking to replicate the study.

3-2. Our scRNA-seq data showed the marker expressions for fibroblasts, adipocytes, pericytes, and osteoblasts in the muscle cluster (new Supplementary Fig. 7), as well as neural progenitors, astrocytes and oligodendrocytes in the neural cluster (new Supplementary Fig. 8). The SAA-TUJ1- cells can be any of these cell types.

To assess the proportion of TUJ1+ cells, we subdivided the neural cluster into mature neural cells (predominantly TUJ1+) and neural progenitors (predominantly TUJ1-). The proportion of mature neural cells was 20-60%, depending on the protocol, with 3D protocols generally yielding a higher proportion of mature neural cells (new Supplementary Fig. 8d).

3. Vascularization of bovine muscle organoids was shown by the presence of VE-cad+ cells. What endothelial subtype was generated? Are these endothelial cells arterial or venous?

3-3. Both arterial and venous markers were detected within the endothelial cell cluster in our scRNA-seq data (see Additional Data 3), suggesting that our endothelial cells include several endothelial subtypes.

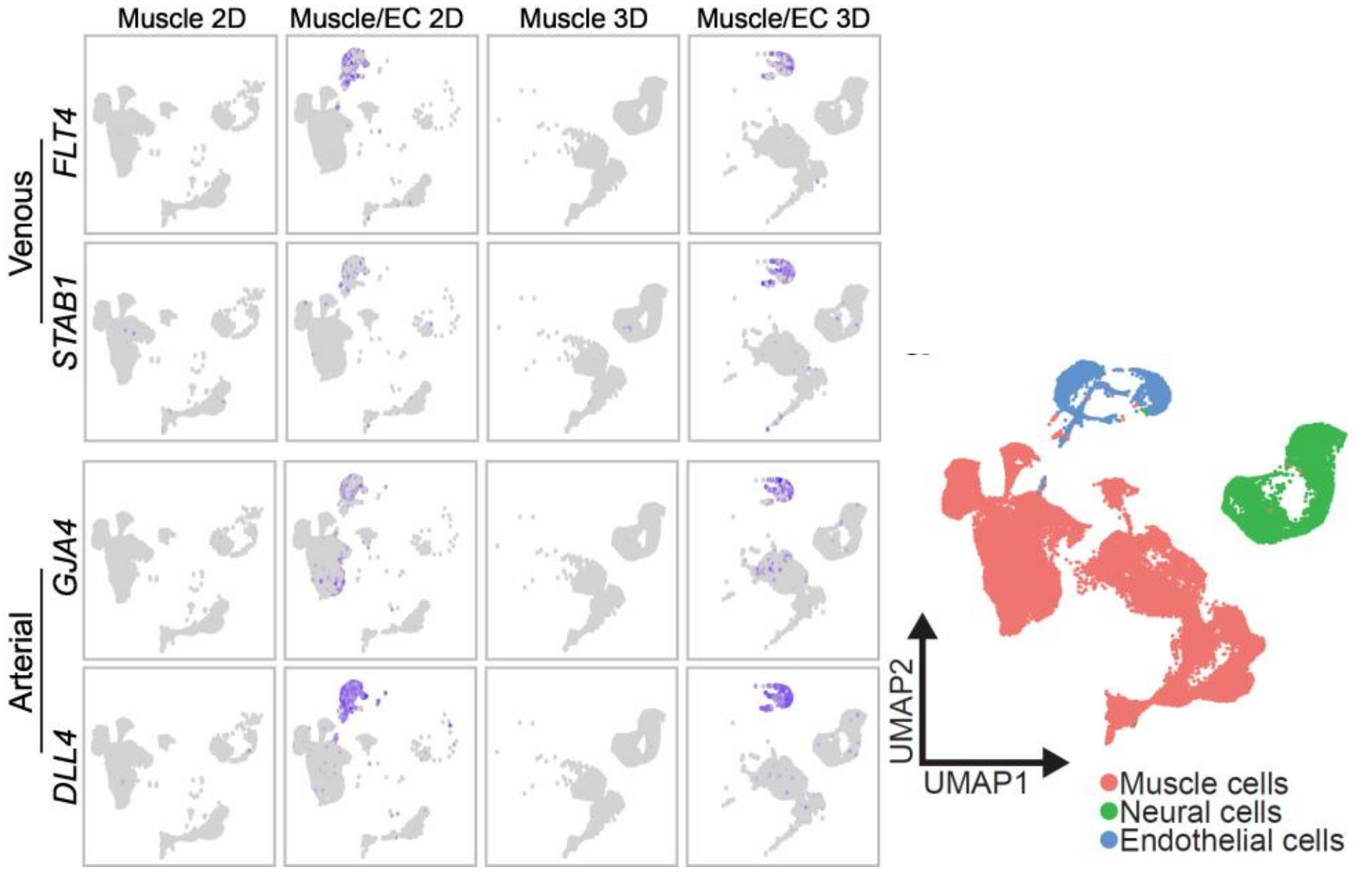


Additional Data 3
UMAP plots colored by the expression of selected marker genes for Venous and Arterial endothelial cells (left). New Fig. 6a is displayed to indicate the endothelial cell cluster (right).

4. The identification of spinal neurons was based on two markers (e.g., TUJ1 and SMI-32). So, it is unclear whether these neurons are indeed “spinal,” as the authors claimed. The authors could assess additional markers to confirm the cellular identity of the neurons. For markers of spinal (motor) neurons, please see https://www.nature.com/articles/s41467-022-35574-x

3-4. This is an important point. We examined spinal (motor) neuron markers in our scRNA-seq data (new Supplementary Fig. 8i). While some markers, such as FOXP1 and HOXC6, were clearly expressed in the neural cluster, others, like ISL1, exhibited only weak expression. Given these data and the relatively high proportion of neural progenitors (new Supplementary Fig. 8d), we toned down our claim by removing the term 'spinal' in several sentences and highlighting the heterogeneous nature of neural subtypes.

5. In the discussion section, elaborating on how the mixed signals used to make two cell types would provide deeper insights into how the cells were generated.

3-5. Our strategy was to combine muscle induction factors (HGF, IGF, and bFGF) with endothelial induction factors (VEGF and forskolin) and then optimize the timing and duration of the treatments. In the manuscript, we explained that "we integrated our bovine muscle induction protocol with established protocols for human endothelial cells50–52, by treating PSM cells with the angiogenic factors VEGF and forskolin (cAMP signaling activator) together with the skeletal muscle induction factors HGF, IGF, and bFGF (Fig. 3a)".

6. Finally, since skeletal muscle, endothelial, and neuronal cells were generated from two different protocols, it would be beneficial for the authors to address how future work needs to integrate these cell types into a single culture system.

3-6. We apologize for the misleading text. Even under the muscle/EC co-induction protocols, TUJ1-positive neurons were already present (new Supplementary Fig. 3; 5a). Our scRNA-seq analyses also detected neural clusters in both 2D and 3D cultures under the muscle/EC co-induction protocols (new Fig. 6b,c; new Supplementary Fig. 8). Note, however, that the proportion of neural cells was lowest under the muscle/EC 2D protocol. We modified the manuscript to explicitly mention the existence of neurons under the muscle/EC protocols.

We thank all the reviewers for their constructive comments.

**In reply to Reviewer 1:**

The authors have performed additional experiments to characterize their in vitro models further. However, several critical concerns remain unresolved and must be addressed before the manuscript can be considered for publication in Nature Communications. Unfortunately, the revised version does not provide sufficient evidence to support the main conclusions, raising significant concerns.

- In the abstract, the authors claim that neurons and muscles self-organize in their model. However, based on the provided immunofluorescence images, there is no clear evidence that the 3D structures self-organize. The neurons and muscles appear intermixed without signs of spatial organization. The authors should revise their claim to reflect their findings more accurately or provide compelling evidence of distinct compartmentalization.

1-1. By "the self-organization of tissue structures" in the abstract, we meant the formation of vessel-like endothelial networks rather than the spatial organization of neural and muscle cells. Nevertheless, we appreciate the reviewer's point and have therefore revised this wording to "the formation of tissue-like structures".

For the same reason, we have replaced the term "self-organization" with "formation" throughout the remainder of the manuscript, except in cases where "self-organization" specifically refers to endothelial/vascular structures, or is used in the context of future directions.

- Insufficient Evidence for Neuromuscular Junction (NMJ) Formation (Lines 114–116). The authors report that blocking calcium transients in muscle cells is sufficient evidence to prove NMJ formation. We strongly disagree with this conclusion. α-Bungarotoxin (α-BTX) staining is a well-established and widely accepted marker of postsynaptic acetylcholine receptor (AChR) clustering, which is critical for confirming NMJ formation. The absence of α-BTX staining suggests that functional NMJs have not fully developed in this model, as AChRs are likely not clustering at the postsynaptic membrane. If presynaptic terminals fail to make stable contact with muscle fibers, postsynaptic differentiation (AChR clustering) will not occur. Curare alone is not sufficient to prove NMJ formation unless there is additional evidence of AChR clustering. To address this, the authors must: Provide α-BTX staining in the revised version. If NMJs are not convincingly demonstrated, the authors should revise their claims to indicate the presence of neurons and muscle cells rather than functional NMJs. In the discussion, the authors may state that future work will focus on optimizing conditions for NMJ formation.

1-2. We thank the reviewer for this helpful suggestion. Accordingly, we performed α-BTX staining and observed punctate signals in contact with TUJ1-positive neurites (new Fig. 2i).

The α-BTX-positive puncta displayed a round and relatively simple morphology, similar to NMJs induced from human iPSCs (e.g., Fig. 5 in Faustino Martins et al., Cell Stem Cell, 2020; doi:10.1016/j.stem.2020.08.011), suggesting that the NMJs generated in our induction system may be immature.

In the discussion, we also stated that "for the maturation of the neuromuscular junctions, further optimization of the induction protocol and longer-term culture may be required".

- Additionally, the manuscript does not justify why cultures are only maintained for 15 days. It is possible that this timeline is too short for NMJs to mature. The authors should

discuss whether longer culture durations might improve NMJ formation. Are the authors observing the formation of a necrotic core and for that reason they can not maintain the 3D cultures longer? If yes, this information should be included in the manuscript.
1-3. To address this point, we extended the Muscle 3D cultures up to day 30. As shown in the z-stack movie, no necrotic core was observed within the aggregates (Additional Video 1.mp4). Because no appreciable differences in aggregate size or skeletal muscle proportion were observed between day 15 and day 30 cultures, we focused on the shorter culture duration, which is also more cost-effective.

- The requested analysis of individual experiments reveals batch effects that influence the curare results. It seems that only one experiment showed some contraction (exp2) but not the other two experiments (exp1 and exp3). The authors should provide separate data for each batch with and without curare treatment to improve clarity and reproducibility. Please clearly discuss any batch-to-batch variability observed in your experiments. Unfortunately, these data are not convincing to support the formation of NMJs in the model.
1-4. To address reproducibility of the curare results, we repeated the same experiments two additional times (new Fig. 2c). Although the frequency of calcium oscillations varied across batches and regions of interest (ROIs), a consistent difference was observed between curare-treated samples and untreated controls. To more clearly illustrate the batch-to-batch variability, here we provide the results from individual experiments (Additional Data 1).

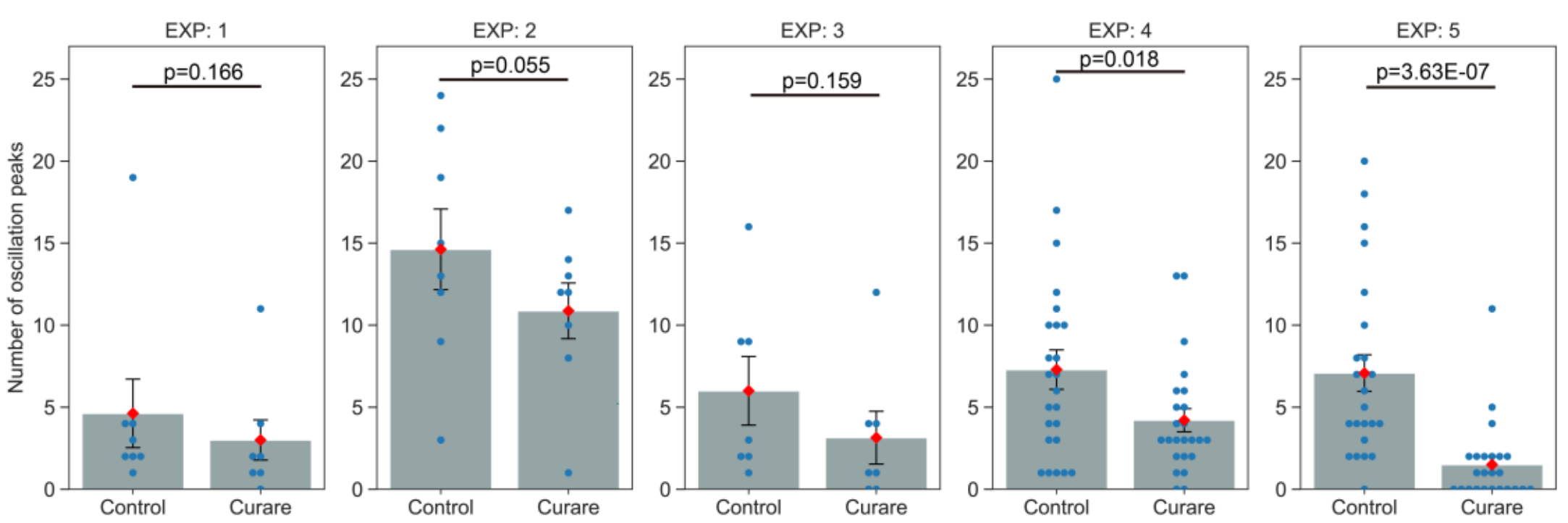


Additional Data 1
Quantification of the total number of calcium oscillation peaks within 2 min in the absence and presence of curare. Data from 5 independent experiments are shown. Mean ± SEM. P-value is from paired t-test. Combined data are shown in new Fig. 2c.

- The authors must clarify whether their neurons are spinal cord motor neurons before making conclusions about their differentiation pathway (Lines 120-122). The authors do not provide evidence that their neurons possess a spinal cord motor neuron identity.They must use specific Hox markers and immunofluorescence analysis to support their claims to determine whether their neurons have a spinal cord or hindbrain identity. If the neurons originate from neuromesodermal progenitors, they should acquire a spinal cord identity. If the immunofluorescence analysis reveals hindbrain identity, the authors must revise

their claims in Lines 120–122 and remove the assertion that their neurons originate from NMPs.

1-5. Following the suggestion, we performed HOXC6 staining and observed nuclear signals in NEFM-positive neurons (new Supplementary Fig. 9j). We also detected expression of *HOXC4*, *HOXC5*, *HOXC6*, *FOXP1*, and *ISL1* in the neural cell cluster of the scRNA-seq dataset (new Supplementary Fig. 9i). These results are consistent with spinal cord-level identity.

Because these markers were not expressed in all neurons, however, we tempered the interpretation in the manuscript to state “These neurons may be, at least in part, spinal neurons derived from neuromesodermal progenitors (NMPs)”.

- The quantification in Figure 6c summarizes the presence of neural, endothelial, and muscle cells from single-cell RNA sequencing data. However, it is concerning that smooth muscle cells appear highly prominent in the 2D and 2D/EC models, while the skeletal muscle cluster is a minor population. Since the goal is cultured beef production, the focus should be on skeletal muscle development rather than smooth muscle. To improve clarity, the authors must plot the percentage of smooth muscle vs. skeletal muscle in each model. Please clearly distinguish between skeletal and smooth muscle cells for each condition in the revised version.

1-6. We agree with the reviewer that this is an important point for the conclusions of this study. We therefore performed staining for smooth muscle (α-SMA) and skeletal muscle (MYOG and SAA) markers across four models (Muscle 2D, Muscle/EC 2D, Muscle 3D, and Muscle/EC 3D) (new Supplementary Fig. 8). The proportions revealed by staining were consistent with those estimated from the scRNA-seq data: in the 2D models, a substantial fraction of smooth muscle was detected, whereas in the 3D models, skeletal muscle was more predominant than smooth muscle.

We thus stated that “the skeletal muscle-rich 3D cultures are more suitable for cultured beef applications than the 2D cultures”.

Because accurate quantification of cell-type proportions is challenging using staining alone, we rely more on the scRNA-seq-based estimates and have therefore moved these data from the Supplementary Information to the main figures (new Fig. 6d).

- Thank you for providing a video of beating 3D cultures. However, the contractions are

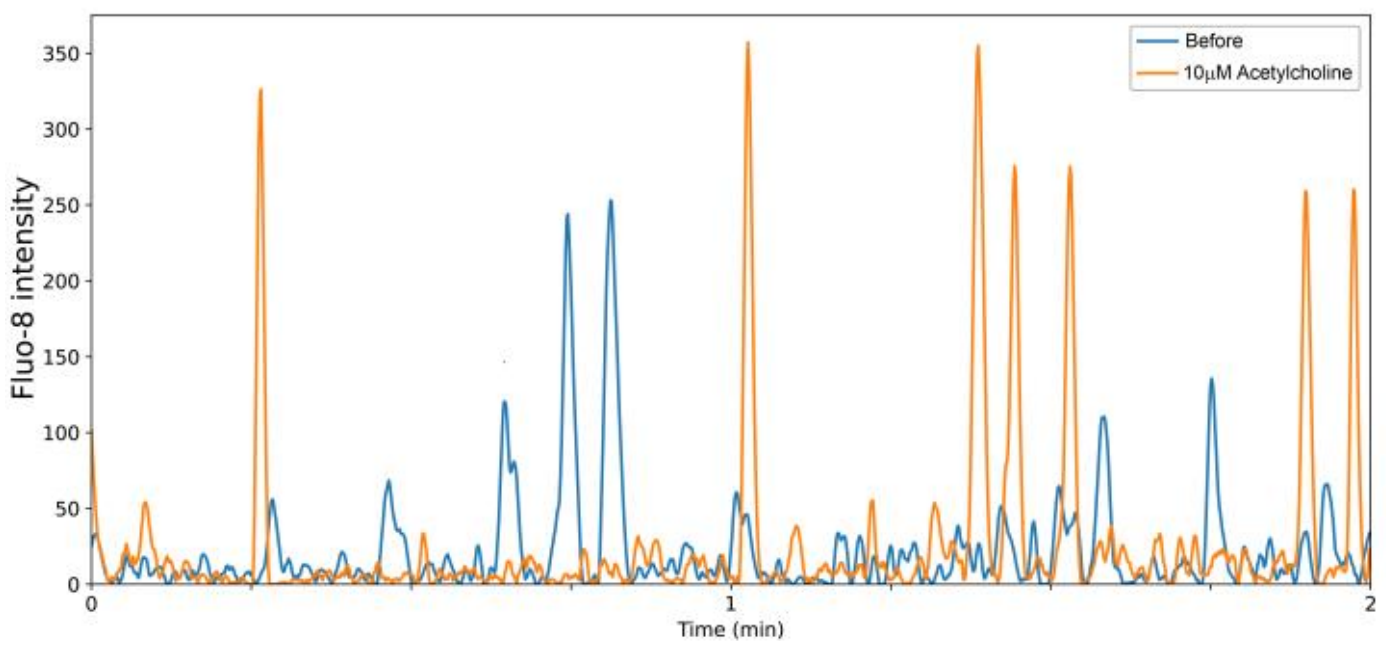


Additional Data 2
Calcium level fluctuations in the absence (blue) and presence (orange) of 10 μM acetylcholine.

only occasionally observed and are not clearly defined. To strengthen their findings, the authors could: (i) Induce contractions via acetylcholine stimulation to determine if neuromuscular activity can be enhanced. (ii) Improve video documentation to clearly illustrate contraction events.

1-7. As noted by the reviewer, muscle contractions were observed only occasionally, whereas calcium oscillations were detected more consistently.

In response to the reviewer's suggestion, we also tried acetylcholine stimulation. Although acetylcholine increased the frequency of calcium oscillations (Additional Data 2), it did not produce a clear or consistent enhancement of muscle contraction.

Therefore, we chose not to describe muscle contraction in the manuscript.

- To improve the manuscript's impact, the authors should clearly state which of their models is the most suitable for cultured meat production based on:

a) Muscle content/yield

b) Cost-effectiveness

c) Reproducibility

This would help guide future applications of their work in meat production.

1-8. Given the higher proportion of skeletal muscle, 3D protocols appear more suitable for cultured meat production than 2D protocols, as discussed in point 1-6.

The choice between Muscle 3D and Muscle/EC 3D protocols depends on the purpose: if endothelial cells are required, Muscle/EC 3D should be used.

The current costs render both the Muscle 3D and Muscle/EC 3D protocols commercially unfeasible, as stated in the discussion.

The protocols are highly reproducible: among the N = 57 samples generated using the Muscle 3D protocol (used in new Fig. 4g), all samples formed aggregates and exhibited an SAA/DAPI staining ratio of at least 10%, corresponding to 100% reproducibility. When a more stringent threshold of 20% SAA-positive staining was applied, 88% of the aggregates met this criterion.

These points are addressed in the manuscript.

- The authors should acknowledge in their discussion that their bovine 3D meat cultures currently rely on ECM derived from mouse carcinoma cells. While this may be necessary for now, future efforts should focus on developing an alternative, animal-free ECM for commercial viability and regulatory approval.

1-9. We completely agree and stated that "Matrigel, which is derived from mouse carcinoma cells, should be replaced with animal-free alternatives" in the discussion.

By addressing these concerns, the manuscript will be significantly stronger, more rigorous, and better aligned with its claims.

We are grateful to the reviewer for their constructive feedback.

**In reply to Reviewer 2:**

I co-reviewed this manuscript with one of the reviewers who provided the listed reports. This is part of the Nature Communications initiative to facilitate training in peer review and to provide appropriate recognition for Early Career Researchers who co-review manuscripts.

**In reply to Reviewer 3:**

The authors have addressed most of my comments in their revision. Adding single-cell RNA sequencing data to quantify cell type proportions significantly strengthens the manuscript and provides additional definitive insights into differentiation outcomes. I have no further major comments.

Remaining minor concerns:

The expression of the pericyte marker of KCNJ8 and ABCC9 overlaps with the expression of smooth muscle-specific markers (MYH11), suggesting that the population could be smooth muscle cells (Supplemental Figure 7i).

3-1. To distinguish smooth muscle cells from pericytes, we performed immunostaining for the pericyte marker NG2 (new Supplementary Fig. 8c). NG2 staining revealed punctate staining signals in contact with VE-cad-positive endothelial cells, supporting the presence of pericytes.

The manuscript would benefit from a more thorough discussion regarding the reproducibility of the described protocols across different bovine embryonic stem cell lines.

3-2. The protocols are highly reproducible: among the N = 57 samples generated using the Muscle 3D protocol (used in new Fig. 4g), all samples formed aggregates and exhibited an SAA/DAPI staining ratio of at least 10%, corresponding to 100% reproducibility. When a more stringent threshold of 20% SAA-positive staining was applied, 88% of the aggregates met this criterion.

Unfortunately, we have tested only a single bovine ES cell line (established in Bogliotti et al, PNAS, 2018; doi: 10.1073/pnas.1716161115), primarily due to the limited availability of high-quality bovine pluripotent stem cell lines. The challenges and current status of bovine pluripotent stem cells are well summarized in a recent review (Chen et al., Int J Mol Sci, 2024; doi: 10.3390/ijms25042120). We stated that "As the protocols were developed using a single bovine ESC line, evaluation with independent sources of bovine pluripotent stem cells will be important" in the discussion.

The scRNA-seq analysis reveals a predominance of smooth muscle cell populations, with a relatively smaller fraction expressing skeletal muscle cell markers. This suggests a deviation from the original objective of generating skeletal muscle cells. I recommend the authors conduct further analysis of their existing data and/or discuss this observation that future protocols can further enhance skeletal muscle differentiation efficiency. This would better align the results with the overall goals of the study.

3-3. We agree with the reviewer that this is an important point for the conclusions of this study. We therefore performed staining for smooth muscle (α-SMA) and skeletal muscle (MYOG and SAA) markers across four models (Muscle 2D, Muscle/EC 2D, Muscle 3D, and Muscle/EC 3D) (new Supplementary Fig. 8). The proportions revealed by staining were consistent with those estimated from the scRNA-seq data: in the 2D models, a substantial fraction of smooth muscle was detected, whereas in the 3D models, skeletal muscle was more predominant than smooth muscle.

We thus stated that “the skeletal muscle-rich 3D cultures are more suitable for cultured beef applications than the 2D cultures”.
Because accurate quantification of cell-type proportions is challenging using staining alone, we rely more on the scRNA-seq-based estimates and have therefore moved these data from the Supplementary Information to the main figures (new Fig. 6d).

We are grateful to the reviewer for their constructive feedback.

**Point-by-point response**

**In reply to Reviewer 1:**

In this manuscript, Sanaki-Matsumiya et al. present a protocol for the co-induction of skeletal muscle, neuronal, and endothelial lineages from bovine embryonic stem cells, resulting in the formation of multicellular 2D and 3D tissue-like structures. The study is technically interesting and relevant to both organoid biology and cultured meat applications. The use of serum-free conditions, the relatively rapid differentiation, and the inclusion of single-cell RNA sequencing add significant value to the work. The revised manuscript has improved in several aspects, including additional staining, functional assays, and a more transparent analysis of cell-type composition. However, concerns remain regarding the interpretation of neuromuscular junction formation, functional maturity, and reproducibility. Addressing these points would further strengthen the manuscript and better align the conclusions with the data.

We thank the reviewer for recognizing the technical interest of our work and its relevance to organoid biology and cultured meat applications.

Major comments

The evidence for neuromuscular junction (NMJ) formation remains limited. While the addition of α-bungarotoxin staining and expanded calcium imaging experiments is appreciated, the data do not yet convincingly demonstrate fully functional NMJs. The α-BTX signal appears as punctate staining without quantitative analysis of acetylcholine receptor clustering or clear co-localization with presynaptic markers. In addition, curare-sensitive calcium oscillations, while suggestive, are not sufficient on their own to establish functional neuromuscular synapses, and muscle contractions remain inconsistent and were not included. Given that α-BTX staining can also produce non-specific punctate background, the interpretation is further complicated. It would therefore be appropriate to temper the claims and refer to these structures as early NMJ-like interactions. Quantification of α-BTX-positive clusters, ideally correlated with functional readouts such as curare response in the same samples, would substantially strengthen the conclusions.

1. Following the reviewer's suggestion, we now refer to these α-BTX-reactive structures as "early NMJ-like" rather than functional NMJs throughout the revised manuscript. We have also explicitly highlighted "these puncta exhibited a rounded, immature morphology" and noted that "further functionalization and maturation of the blood vessels and early NMJ-like structures will be necessary".

We also quantified α-BTX staining (new Fig. 2j; new Supplementary Fig. 3b-d). The α-BTX-reactive spots were classified based on their proximity to muscle fibers and neurons. Early NMJ-like: within 1 µm of both muscle fibers and neurons. Muscle-associated: within 1 µm of muscle fibers but not neurons. Neuron-associated: within 1 µm of neurons but not muscle fibers. Orphaned: farther than 1 µm from both muscle fibers and neurons. As the α-BTX intensity distribution was bimodal, only spots with intensities above the threshold (1298) were included in subsequent analyses (new Supplementary Fig. 3c). After normalizing to the areas of muscle fibers, neurons, overlapping regions (early NMJ-like), and non-specific regions (orphaned), the density of early NMJ-like spots was significantly higher than that of the other categories (new Fig. 2j). These results demonstrate that α-BTX-reactive spots are frequently located at sites where muscle fibers

and neurons were in close proximity, supporting the presence of early NMJ-like structures at neuron-muscle contact sites.

The additional experiments addressing curare sensitivity are helpful, but they reveal notable inter-batch variability. The reported consistent differences between treated and untreated samples are not uniformly evident across all experiments, and this variability is largely obscured in the summarized presentation. Presenting the batch-wise data more prominently would improve transparency. In addition, the statistical approach should be more clearly described and justified, particularly with regard to the treatment of biological replicates versus regions of interest and the handling of inter-batch variability. A brief discussion of possible sources of variability, such as differences in differentiation efficiency, neuronal density, or maturation state, would also be important.
2. Following the reviewer's suggestions, we now present batch-wise data in new Supplementary Fig. 3a to improve transparency regarding inter-batch variability. As the reviewer pointed out, calcium activity patterns varied substantially among individual cells, even within the same batch. In contrast, the effect of curare was more discernible when responses were compared before and after treatment within the same ROI (namely, the same cell). Therefore, we consider the paired t-test to be the most appropriate statistical method for this analysis.

We have also revised all figure legends to explicitly indicate whether N represents ROIs, cells, images, aggregates, or dishes, as appropriate, in addition to the number of independent experiments for each analysis. Regarding the potential sources of variability, we discussed that "The frequency and amplitude of these calcium transients varied among cells, potentially reflecting differences in cell density and maturation state".

The extension of cultures to day 30 without evidence of necrosis is informative; however, the lack of observable maturation between day 15 and day 30 raises important questions about the developmental potential of the system. Early NMJs do not appear to mature over time, and muscle contractions remain weak and inconsistent. The manuscript would benefit from a more explicit discussion of the factors that may limit maturation in this system, such as the absence of activity-dependent cues, appropriate chemical signals, Schwann cells, etc., and how these limitations affect the interpretation of the model.
3. Following the reviewer's suggestion, we explicitly mentioned that "However, we did not observe a clear positive impact of bovine endothelial networks and neurons on muscle cultures, even after 30 days of culture" and stated that "further maturation of the early NMJ-like structures may require optimization of the induction protocol and extrinsic signaling cues, as well as the incorporation of additional supporting cell types, such as Schwann cells, during long-term culture".

The characterization of neuronal identity has been strengthened by the inclusion of HOX gene expression and motor neuron markers. However, the HOXC6 immunostaining does not appear exclusively nuclear, which raises some uncertainty regarding its interpretation. Clarifying this point or further tempering the statement would improve the manuscript.
4. We thank the reviewer for this observation. We agree that the HOXC6 immunostaining pattern in our samples was not exclusively nuclear. However, similar nuclear and

cytoplasmic HOXC6 staining patterns have been reported in previous studies (e.g., SMH Li et al, PMID: 40117347, see Fig 4E; P Malvi et al, PMID: 37951219, see Fig. 1B).

To further address this point, we quantified the immunostaining data and found that 70% of the HOXC6 signal overlapped with the nuclear compartment, supporting its nuclear enrichment (new Supplementary Fig. 10k).

The single-cell RNA sequencing analysis and additional staining reveal a substantial contribution of smooth muscle cells, particularly in 2D conditions, with skeletal muscle being less dominant. While the authors appropriately highlight that 3D cultures are more favorable, this observation points to limitations in lineage specificity and control within the differentiation protocol. A more explicit discussion of the biological basis for the differences between 2D and 3D conditions would help explain the observed differences, as well as potential strategies to improve skeletal muscle specification. This also raises concerns regarding the current suitability of the system for skeletal muscle-focused applications.

5. Following the reviewer's suggestion, we stated that "However, they also present the challenges of precisely controlling cell differentiation processes: variations in protocols, such as 2D versus 3D cultures, can lead to unexpected differences in cell type composition, potentially due to differences in substrate and tissue stiffness, cell density, and other microenvironmental cues that influence cell fate decisions".

Given the broader muscle composition observed, the authors may wish to consider whether the current title fully reflects the scope of the study, and whether replacing "skeletal muscle" with "muscle" would provide a more accurate description.

6. Following the reviewer's suggestion, we have replaced "skeletal muscle" with "muscle" in the title and abstract.

Finally, the use of a single bovine ESC line limits generalizability, and although this is acknowledged, it remains an important consideration related to the reproducibility and scalability of the approach.

7. We have tested only a single bovine ES cell line primarily due to the limited availability of high-quality bovine pluripotent stem cells. We further emphasized this limitation in the manuscript, stating that "As the protocols were developed using a single bovine ESC line, evaluation with independent sources of bovine pluripotent stem cells will be important for reproducibility and scalability".
