# Supplementary material for: Self-organization of vascularized muscle from bovine embryonic stem cells
Source: Nat Commun. 2026 Sep 2;17:8975. doi: 10.1038/s41467-026-76569-2 (PMC13538390; doi:10.1038/s41467-026-76569-2)
Supplement: Supplementary file 4 [file 41467_2026_76569_MOESM3_ESM.pdf]

Description of Additional Supplementary Files

- Supplementary Data 1

Size and cost of muscle aggregates.

- Supplementary Data 2

Primer sequences for qRT-PCR.

- Supplementary Data 3

Summary of the scRNA-seq statistics.

- Supplementary Movie 1

Time-lapse imaging of calcium level fluctuations in the day-18 culture of the muscle 2D protocol using Fluo-8 in the absence (left) and presence (right) of 10 μM curare. The time stamp indicates min:sec. The snapshot is also shown in Fig. 2a. Images were taken with the Thunder Imager Live Cell & 3D assay.

- Supplementary Movie 2

Z-stack IHC images of the day-15 aggregates of the muscle 3D protocol. SAA and TUJ1 are muscle fiber and neuronal markers, respectively. Z-stack interval is 2 μm. The snapshot is also shown in Fig. 4d. Images were taken by a MuVi-SPIM Light-sheet microscope.

- Supplementary Movie 3

Z-stack IHC images of the day-15 aggregate of the muscle/EC 3D protocol. SAA and VE-cad are muscle fiber and endothelial markers, respectively. Z-stack interval is 2 μm. The snapshot is also shown in Fig. 5d. Images were taken by a MuVi-SPIM Light-sheet microscope.

- Supplementary Movie 4

Z-stack IHC images of the day-15 aggregate of the muscle/EC 3D protocol. ZO-1 is a tight junction marker. Z-stack interval is 2 μm. The max projection is also shown in Fig. 5g. Images were taken by a MuVi-SPIM Light-sheet microscope.

- Supplementary Movie 5

Z-stack IHC images of the day-15 aggregate made from the PSM cells that were induced without BMP inhibitor DMH1. Z-stack interval is 2 μm. The snapshot is also shown in Supplementary Fig. 6b. Images were taken by a MuVi-SPIM Light-sheet microscope.
